# Electrochemistry as a Tool for Redox‐Based Bio‐Information Processing

**DOI:** 10.1002/advs.202510184

**Published:** 2025-08-22

**Authors:** Eunkyoung Kim, Chen‐yu Chen, Fauziah Rahma Zakaria, Dana Motabar, Mijeong Kang, Deanna L. Kelly, Alessandra Napolitano, William E. Bentley, Gregory F. Payne

**Affiliations:** ^1^ Institute for Bioscience and Biotechnology Research University of Maryland College Park Maryland 20742 USA; ^2^ Robert E. Fischell Institute for Biomedical Devices University of Maryland College Park Maryland 20742 USA; ^3^ Fischell Department of Bioengineering University of Maryland College Park Maryland 20742 USA; ^4^ Department of Optics and Mechatronics Engineering Pusan National University Busan 46241 Republic of Korea; ^5^ Maryland Psychiatric Research Center University of Maryland School of Medicine Baltimore MD 21228 USA; ^6^ Department of Chemical Sciences University of Naples Federico II Via Cintia 4 Naples I‐80126 Italy

**Keywords:** electrochemiluminescence, electrogenetics, mediated electrochemistry, melanin, oxidative stress, redox biology, spectroelectrochemistry

## Abstract

Redox, a native modality in biology involving the flow of electrons, energy, and information, is used for energy‐harvesting, biosynthesis, immune‐defense, and signaling. Because electrons (in contrast to protons) are not soluble in the medium, electron‐flow through the redox modality occurs through redox reactions that are sometimes organized into pathways and networks (e.g., redox interactomes). Redox is also accessible to electrochemistry, which enables electrodes to receive and transmit electrons to exchange energy and information with biology. In this Perspective, efforts to develop electrochemistry as a tool for redox‐based bio‐information processing: to interconvert redox‐based molecular attributes into interpretable electronic signals, are described. Using a series of Case Studies, how the information‐content of the measurements can be enriched using: diffusible mediators; tuned electrical input sequences; and cross‐modal measurements (e.g., electrical plus spectral), is shown. Also, theory‐guided feature engineering approaches to compress the information in the electronic signals into quantitative metrics (i.e., features) that can serve as correlating variables for pattern recognition by data‐driven analysis are described. Finally, how redox provides a modality for electrogenetic actuation is illustrated. It is suggested that electrochemistry's capabilities to provide real‐time, low‐cost, and high‐content data in an electronic format allow the feedback‐control needed for autonomous learning and deployable sensing/actuation.

## Graphical abstract

Redox is a modality that is native to biology and accessible to electronics through electrochemistry. Imposed electronic inputs are transduced into redox signals that can probe for bio‐information or actuate biological responses. Measured electronic output responses are analyzed to reveal actionable information. It is envisioned that electrochemistry can bring the power of electronics to redox biology.

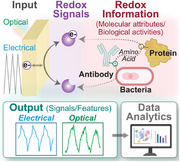

## 1 Redox in Biology

**Figure 1:**
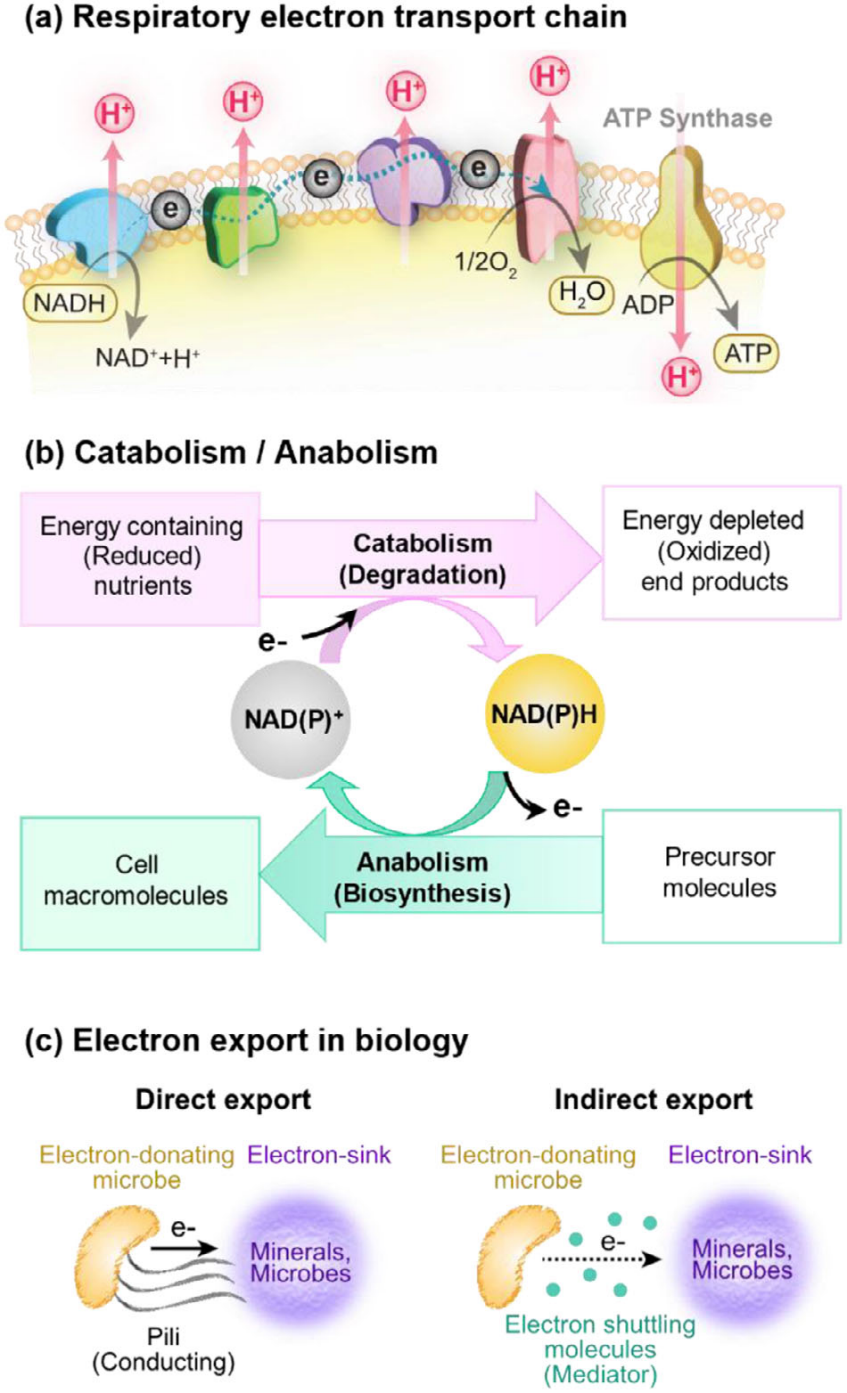
Familiar biological redox activities in bioenergetics and biosynthesis. a) Respiratory electron transport chain is integral in bioenergetics in aerobic metabolism. b) Diffusible electron carriers (NAD(P)H) shuttle electrons between catabolic (e.g., energy‐harvesting) and anabolic (i.e., biosynthetic) pathways. c) Emerging research shows microbes can export electrons into their environment through mechanisms that do not involve O_2_ as terminal acceptor.

Biology uses the redox modality to control the flow of electrons, energy, and information.^[^ ^1^ ^]^ Unlike protons, electrons are not soluble in aqueous media, and thus, “electron‐flow” through the redox modality involves a series of electron‐transfer reduction‐oxidation (redox) reactions. Probably biology's most familiar uses of the redox modality are for bioenergetics and biosynthesis. For instance, **Figure** **1**a shows the respiratory electron transport chain, while Figure 1b illustrates the role of diffusible carriers (e.g., (NAD(P)/NAD(P)H)) that can shuttle electrons between electron‐donating oxidation reactions (common in catabolic pathways) and electron‐accepting reduction reactions (common in anabolic pathways). More recent research has focused on the export of electrons outside the cell^[^ ^2^, ^3^, ^4^ ^]^ with the identification of direct (e.g., conducting)^[^ ^5^, ^6^, ^7^ ^]^ and indirect (i.e., mediator‐based) mechanisms for electron export (Figure 1c).^[^ ^8^, ^9^, ^10^ ^]^

**Figure 2:**
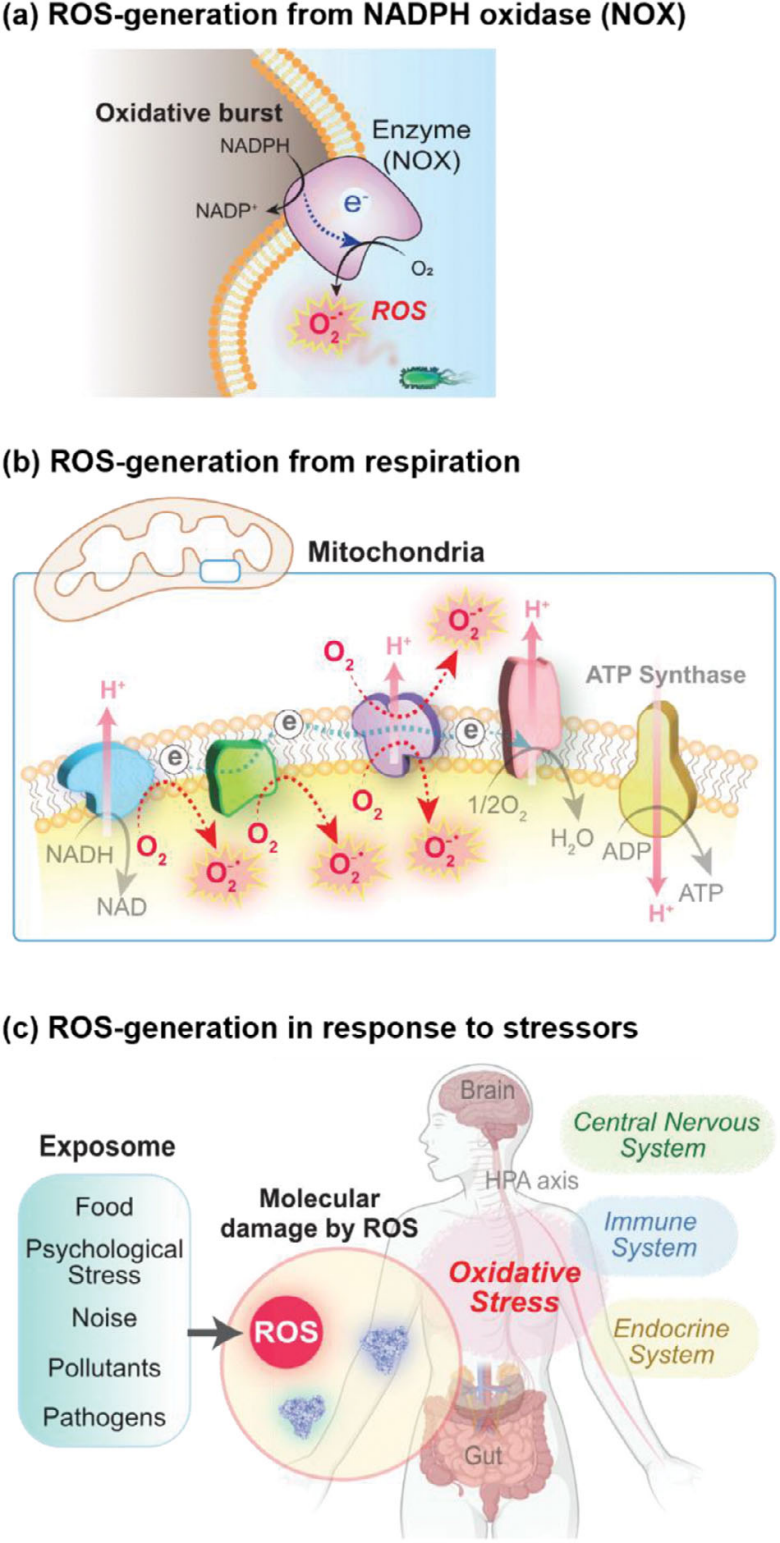
Reactive species (e.g., ROS) are integral to immune responses, oxidative stress, and redox signaling. a) NADPH oxidase (NOX) transfers electrons to O_2_ to generate ROS. b) Electron leakage from the respiratory electron transport chain can lead to ROS‐generation. c) Emerging research suggests that ROS may participate in the systems‐level responses to various stressors.

In addition to bioenergetics, another broad topic in redox biology involves the role of reactive species (e.g., reactive oxygen/nitrogen/chlorine species).^[^ ^11^ ^]^ Historically, reactive species were observed to be generated during an immune response to infection (e.g., a respiratory or oxidative burst; **Figure** **2**a)^[^ ^12^, ^13^ ^]^ and also through electron‐leakage during mitochondrial electron transport (Figure 2b).^[^ ^14^, ^15^, ^16^ ^]^ These reactive species gained further prominence for their proposed roles in health and disease (e.g., the free radical theory of aging and oxidative stress).^[^ ^15^, ^17^, ^18^ ^]^ Although there remains an incomplete understanding of oxidative stress, Figure 2c indicates that it appears to: be a physiological response to external stressors (e.g., psychosocial stress^[^ ^19^ ^]^); span organ systems to operate at a systems level (e.g., hypothalamus‐pituitary‐adrenal axis);^[^ ^20^, ^21^ ^]^ and have important (but ill‐defined) causes and consequences relevant to health and disease.^[^ ^22^, ^23^, ^24^, ^25^ ^]^

From a chemistry perspective, it is important to note that the reactive species (e.g., O_2_^•−^, OH^•^, ONOO^−^, HOCl, and H_2_O_2_) are not identical – but have different reactivities/lifetimes.^[^ ^26^, ^27^ ^]^ Further, the common “targets” of reactive species (e.g., the amino acid residues of proteins) are also not identical, but respond differently to the individual reactive species.^[^ ^27^, ^28^, ^29^ ^]^ Thus, even in the absence of molecular recognition mechanisms, redox reactions appear to offer considerable chemical selectivity. For instance, the post‐translational oxidation of proteins occurs through different mechanisms some of which are believed to be integral to biological function (the sulfur‐switching of cysteine thiols to disulfide bonds^[^ ^30^ ^]^ and the oxidative crosslinking of collagen^[^ ^31^, ^32^, ^33^ ^]^) while others are considered to be the adverse molecular‐level consequences of oxidative stress (e.g., oxidative deamination and tyrosine nitration).^[^ ^34^, ^35^ ^]^

Finally, it is important to recognize the role of reactive species and the redox modality in the flow of information in biology (e.g., redox signaling).^[^ ^30^, ^36^, ^37^ ^]^ In fact, a decade ago, a “redox code” was proposed to characterize the flow of information through the redox modality.^[^ ^38^ ^]^ Importantly, such redox signals can be “recognized” by redox‐responsive master regulators that can actuate biological responses at the level of gene expression.^[^ ^17^, ^22^ ^]^

### 1.1 Redox Reactions can be Nested into Pathways and Networks

**Figure 3:**
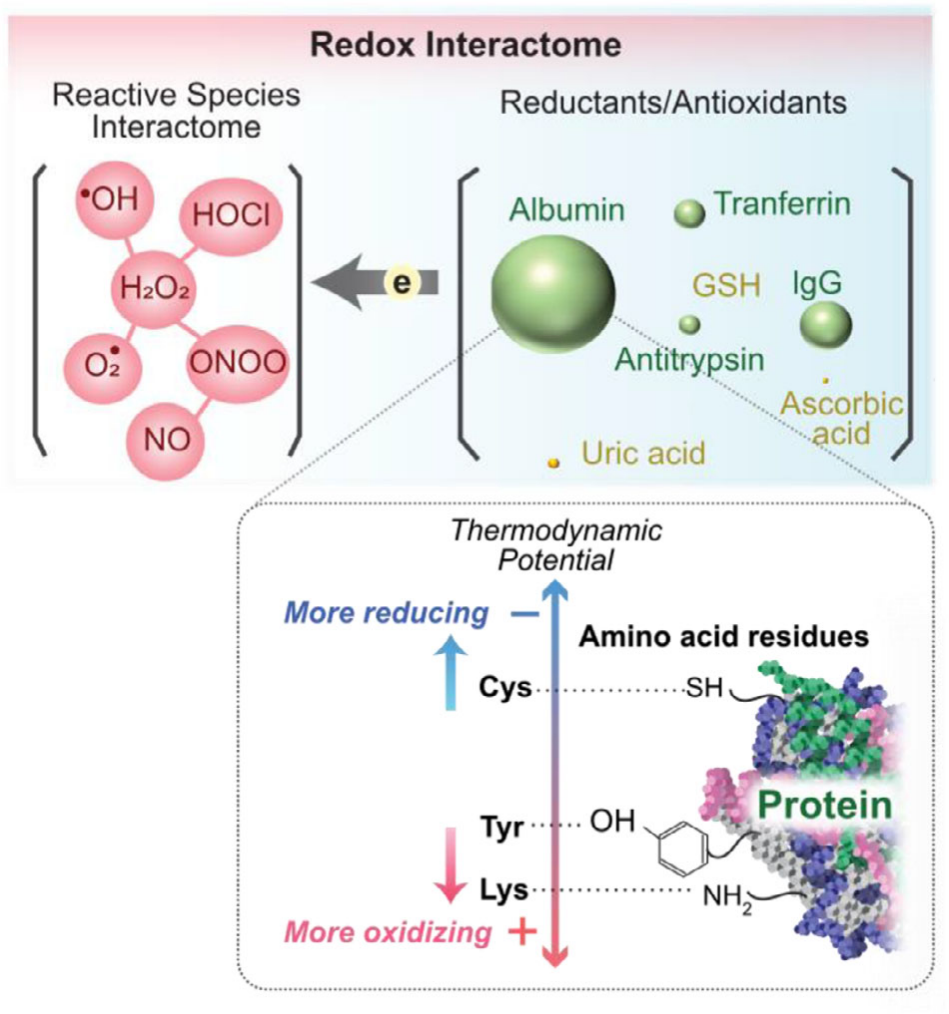
Redox reactions can be nested into networks (i.e., redox interactomes). The reactive species interactome at the left can “extract” electrons from a set of antioxidants. The inset shows that proteins contain various amino acid residues that have different redox potentials and can respond differently (i.e., through different mechanisms) to individual reactive species.

As noted, electrons are insoluble and thus redox interactions involve electron‐transfer reactions that are sometimes organized into pathways (respiratory electron transfer chain) and reaction networks. Such systems‐level network thinking has become especially common for understanding the role of reactive species in signaling and oxidative stress.^[^ ^39^, ^40^ ^]^ For instance, locally‐generated reactive species generally serve as oxidants that extract electrons from local electron‐donors (reductants). In one viewpoint (a signaling perspective), these reactions generate second messengers that can propagate redox information over space/time.^[^ ^41^ ^]^ In an alternative viewpoint (a pathophysiology perspective), these reactions deplete the local environment of reducing‐equivalents making it less resilient to further oxidative insults, which presumably are linked to pathological oxidative stresses.^[^ ^35^ ^]^ These viewpoints tend to organize redox reactions into reaction networks – the reactive species interactome or redox interactome of **Figure** **3**.^[^ ^1^, ^42^, ^43^, ^44^, ^45^ ^]^ This network organization illustrates that reactive species act to induce electron “flow” from a set of redox‐active “nodes” (e.g., reductants).

While Figure 3 is an oversimplification, extracellular redox interactomes can be described as directed networks with nodes that are capable of existing in distinct redox states (often a single reduced state and a single oxidized state). The nodes can be linked (i.e., connected) such that an electron can be transferred from the reduced state of one node to an oxidized state of a “neighboring” node. From a molecular perspective, the redox interactome may include subnetworks. The insert in Figure 3 shows proteins are composed of amino acids residues which have distinctly different redox properties (e.g., cysteine, lysine and tyrosine residues are oxidized through different redox mechanisms to form different products). The direction of all network links is constrained by thermodynamics: electrons “flow” from molecules with more reducing (i.e., more negative) redox potentials to molecules of more oxidizing (i.e., more positive) redox potentials. However, even in the presence of strong thermodynamic driving forces, many redox reactions do not proceed spontaneously (e.g., NOX enzymes are required to catalyze the thermodynamically‐favorable oxidation of NADPH by O_2_). This kinetic “insulation”^[^ ^1^, ^46^ ^]^ is another fundamental difference between redox‐chemistry and acid‐base chemistry (in addition to the difference in solubilities of the H^+^ vs e^−^ charge‐carriers). Specifically, protonation/deprotonation reactions are believed to be rapidly‐reversible such that the protonation states of molecules in a homogeneous compartment are believed to be in dynamic equilibrium, and this state can be characterized by a single pH measurement. In contrast, the redox‐state of a homogenous compartment cannot typically be characterized by a single redox potential measurement, but rather the various redox‐couples in a compartment may exist at different extents of dis‐equilibrium.^[^ ^46^ ^]^ For instance, an enzyme may allow one redox couple to equilibrate while other couples (e.g., NADH/NAD^+^) may exist far from equilibrium. While a redox‐network provides an instructive systems‐level framework that acknowledges that the flow of electrons in biology generally requires redox reactions, our network representation oversimplifies the dynamically‐changing and spatially‐temporally localized set of redox reactions.

In summary, despite the over‐simplifications, abstracting the redox modality in terms of networks provides a convenient systems‐level framework for analysis. This network framework also suggests that the interactome contains important information of biological state and is a potential source for discovering discriminating biomarkers in redox biology.

## 2 Electrochemistry: A Potential Tool For Information Processing

**Figure 4:**
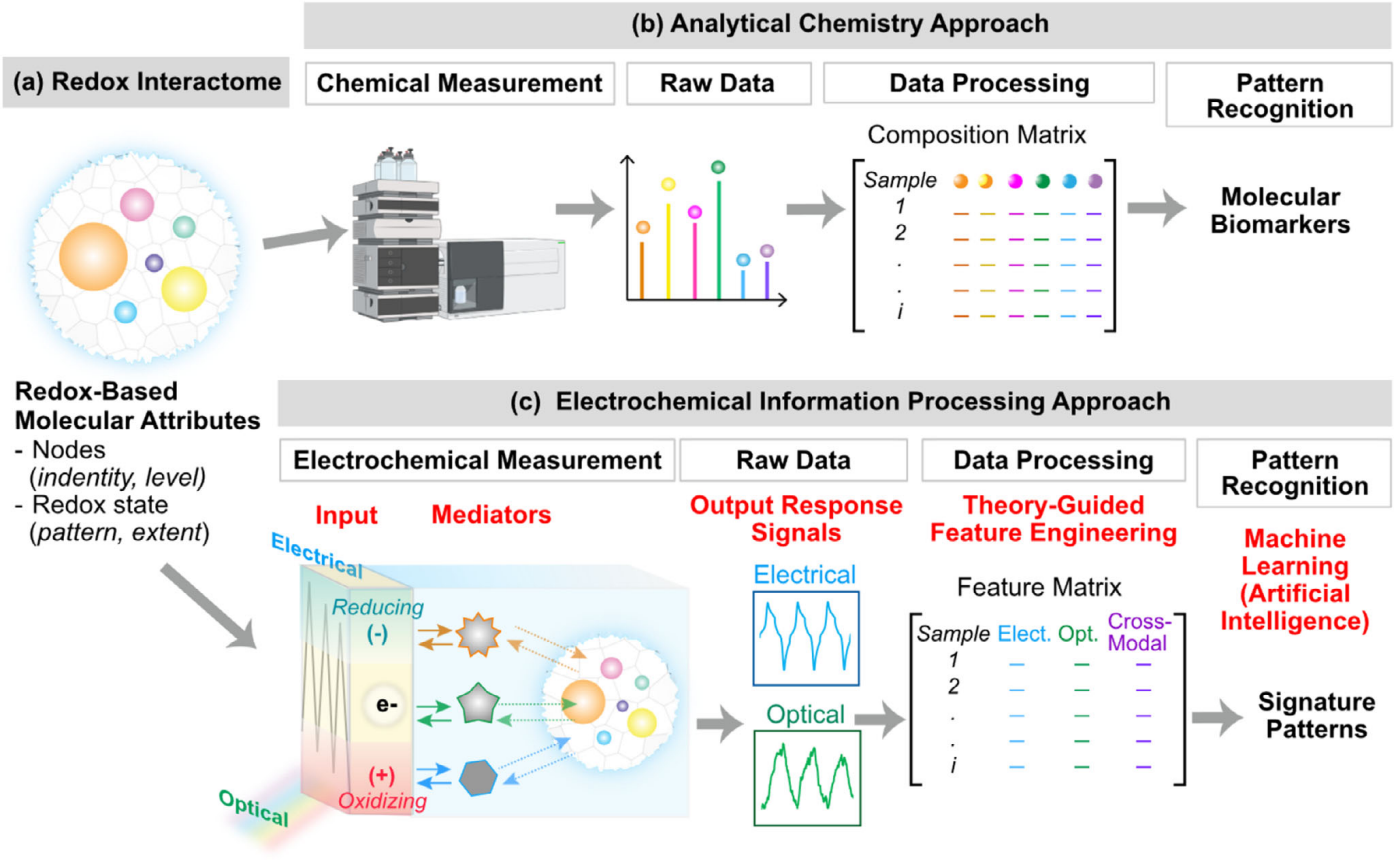
Chemical and information processing approaches to analyze a redox interactome. a) Redox‐based molecular attributes are hypothesized to contain information that can be used to characterize biological state/activities. b) Chemical approaches (e.g., omics) are commonly used to measure an interactome and discern discriminating biomarkers. c) Information processing approach aims to probe an interactome to convert molecular attributes into electronic signals that can discern signature patterns (see text for detail).

**Figure** **4**a illustrates the hypothesis that the molecular attributes of a redox interactome can characterize the state of a complex biological system (e.g., gut or rhizosphere). The redox‐based molecular‐attributes include the set of nodes and the pattern and extent of their oxidation. This hypothesis drives “chemical” approaches (e.g., omics; Figure 4b) to measure the chemical composition and concentrations (i.e., to measure the nodes), and this compositional data can be used to identify a subset of nodes that can serve a discriminating molecular biomarkers.^[^ ^47^, ^48^, ^49^, ^50^ ^]^ When these analytical chemistry approaches succeed, they can provide insights into underlying mechanisms and potential interventions, but these chemical approaches are generally slow, costly, and confined to a few well‐resourced labs. It is important to recognize that some complex biological systems are more‐readily characterized by electrical (not chemical) measurements (e.g., electrocardiograms and electroencephalograms). When electrical approaches succeed, they offer the benefits of speed, simplicity, and portability (e.g., for deployment), but systems‐level electrical measurements often lack the molecular granularity that can reveal mechanisms. We are investigating electrochemical approaches to generate redox‐based electronic signals that can be used to characterize (or change) biological states/activities.

For decades, electrochemistry has been studied as a tool for chemical analysis, and **Table** **1** summarizes its well‐known strengths and weaknesses. The weaknesses – especially the limited molecular‐level selectivity – make it unlikely that electrochemistry will emerge as a generic *tool for chemical analysis* capable of displacing chromatographic methods for resolving a mixture into its individual components. Nevertheless, electrochemical measurements are simple, rapid, sensitive, and miniaturizable, and convert molecular‐information into electronic signals. Thus, Figure 4c shows that we envision using electrochemistry as a *tool for bio‐information processing* such that a redox interactome can be probed to convert the molecular attributes of the interactome into electronic signals that can be analyzed to reveal characteristic signatures of redox states/activities.

**Table 1:** Strengths and weaknesses of electrochemistry for chemical analysis and bio‐information processing.

**Strengths**
Measurements are simple, rapid, inexpensive, sensitive, and miniaturizable. Data is generated in a convenient electronic format.
**Weaknesses**
Redox‐active molecules may be kinetically stable and require overpotentials for reaction (e.g., NADH). Electrical signals often lack molecular‐level selectivity (i.e., it is difficult to deconvolute complex electrical signals into contributions from individual components). Electrical signals may not be stable over time (due to electrode fouling) or between electrodes.

### 2.1 Electrochemical Approaches for Redox‐Based Bio‐Information Processing

Figure 4c illustrates that we are exploring 5 somewhat unconventional electrochemical approaches to convert redox‐based molecular attributes into high‐content electronic signals that can be analyzed to discern biologically‐relevant meaning, or imposed to induce redox‐responsive biological activities. First, we often enlist diffusible mediators to shuttle electrons to/from the electrode to engage our biological system (e.g., to probe a sample's redox‐based molecular attributes; Case Study #1). Typically, as shown in **Table** **2**, we select electrochemical‐mediators (e.g., organometallics) or biological metabolites that are rapidly and reversibly redox‐active, and can readily exchange electrons with the electrode (Tables S1 and S2 (Supporting Information) provide more extensive information and notes on approaches that have been used to overcome inherent limitations of electrochemical measurements). As will be discussed, these mediators can sometimes be selected to target specific redox‐active moieties (e.g., nodes) or redox‐activities (Case Study #3).

**Table 2:** Information on mediators used for redox‐based bio‐information processing.

Mediators	Redox potential V vs Ag/AgCl	Function	Target biomolecules	Refs.
Paraquat (PQ)	−0.64	Reducing	Melanin	[51]
			Dehydroascorbate	[52]
Pyocyanin (PYO)	−0.25	Reducing	Melanin	[53, 54]
		Oxidizing	SoxR	[55]
Ru(NH_3_)_6_Cl_3_ (Ru)	−0.2	Reducing	Melanin	[53, 54]
K_2_Fe(CN)_6_ (Fcn)	+0.17	Oxidizing	Cellular reductants	[55]
Ferrocene dimethanol (Fc)	+0.25	Oxidizing	Melanin	[54, 56]
			Cysteine, methionine residue of antibody	[57, 58, 59]
ABTS^a)^	+0.5	Oxidizing	Melanin	[51]
Acetosyringone (AS)	+0.5	Oxidizing	Melanin	[53, 54]
			OxyR	[60]
K_3_IrCl_6_ (Ir)	+0.55	Oxidizing	Melanin	[61]
			Bio‐reductants (e.g., GSH, albumin, bilirubin, uric acid, ascorbate)	[62, 63]
			Amino acids (e.g., lysine, tyrosine, cysteine, methionine)	[64]
Ru(bpy)_3_Cl_2_ (Ru_b3_^2+^)^b)^	+1.1	Oxidizing	Amino acids (e.g., cysteine, methionine, proline, histidine, tyrosine, tryptophan)	[65]

^a)^ ABTS: 2,2′‐azino‐bis(3‐ethylbenzothiazoline‐6‐sulfonic acid;

^b)^ Ru(bpy)_3_Cl_2_: Tris(bipyridine)ruthenium(II) chloride.

Second, we impose a sequence of electrical inputs at the electrode (e.g., electrical potential inputs; *E*) to controllably‐drive redox reactions at the electrode. [Note: as suggested at the left in Figure 4c, we use the convention of displaying the thermodynamic plots with negative *E*‐values shown at the top to be consistent with a free energy diagram in which electrons/energy spontaneously flow from the top of the scale to the bottom.] For instance, Figure 4c illustrates that when *E* is cycled to be more negative (i.e., more reducing) potentials relative to a mediator's redox potential (*E°*), then electrons flow from the electrode to the mediator to switch its redox state (from oxidized to reduced). When the imposed *E* is cycled to more oxidizing redox potentials, the direction of electron flow is reversed to switch the mediator's redox‐state by extracting electrons from the mediator. As illustrated by the electrical response signals, an oscillating imposed *E* can induce an oscillating flow of electrons to/from the electrode, and this oscillating electrical current can be sensitively measured. As will be discussed, the imposed *E* sequence does not need to be oscillatory, but can be “tuned” to enhance information extraction.

Third, we purposefully increase the information‐content of our measurements. A common goal in conventional electrochemistry is to relate a single measurement metric (e.g., a peak current or steady‐state current) to a concentration, while efforts to increase measurement dimensionality have focused on extending from a single electrode to an electrode array (e.g., common for the artificial nose/tongue).^[^ ^66^ ^]^ We are attempting to increase measurement dimensionality by using multiple mediators, imposing complex potential inputs, and also by simultaneously coupling electrical measurements with measurements from alternative sensor modalities (e.g., optical). Spectroelectrochemical measurements are particularly useful since they can provide complementary electrical measurements of activities (e.g., electrical currents measure reaction kinetics) and spectral measurements of the redox‐state switching of the participating molecular species as illustrated by the optical output responses in Figure 4c.^[^ ^67^, ^68^ ^]^ The richness of spectroelectrochemical measurements occurs because often, the oxidized and reduced states of a molecule have distinguishable spectral differences.

Fourth, we use theory‐guided feature engineering to compress signals into robust quantitative features (i.e., signal metrics) that can summarize the information from high‐content measurements. Sometimes, physicochemical intuition of the underlying mechanisms and domain knowledge of an expert provides the “theory” that guides information‐extraction. For instance, electrochemical measurements are often correlated to concentrations using a single measurable metric (e.g., steady state or peak currents). In other cases, relevant features of the data become more obvious by displaying time‐series results in terms of phase‐plane plots. For instance, time‐series electrochemical measurements are routinely visualized as current‐potential (*i‐E*) phase‐plane plots that reveal information (e.g., of a molecule's redox potentials). In more complicated cases, when measurements are the result of competing physical, chemical or biological phenomena, we have found it useful to rely on a minimal physics‐based deterministic model to reveal the intrinsic structure of the data (see Case Study #2).^[^ ^69^, ^70^ ^]^ An analogy to theory‐guided feature engineering that is familiar to engineers involves characterizing fluid flow. The structure of the data is described by well‐known governing partial differential equations (i.e., Navier Stokes) which often cannot be solved. Yet manipulation of these equations has revealed a quantitative feature (e.g., the Reynolds number) that serves as a correlating parameter for diverse engineering systems. The important point is that the theory (the Navier Stokes equation) suggests how physicochemical variables (density and viscosity) can be combined with systems variables (size/geometry and fluid velocity) to generate a generalizable feature (Reynolds number) that has become integral to understand, predict and design in diverse engineering applications. For theory‐guided feature engineering in bio‐information processing, we envision the governing partial differential equations (of electrochemical reactions and diffusion) can be used to reveal how the data can be reduced into physically realistic features that serve as correlating variables for machine learning (ML). Potentially, theory‐guided feature engineering will allow modeling with lower data‐needs compared to either parameterizing a digital replica or requiring artificial intelligence (AI) methods to learn chemistry.

Finally, we envision that quantitative signal features will serve as variables for data‐driven pattern recognition by ML and AI (see Case Studies #4 and #5). This approach can be viewed as a hybrid between traditional electrochemical modeling of reaction diffusion mechanisms to generate digital replicas, and conventional data‐driven chemometric modeling to identify patterns. In fact, most recent reports applying ML and AI in electrochemistry use such a hybrid approach (theory‐guided plus data‐driven) to efficiently interpret high‐dimensional electrochemical measurements.^[^ ^71^, ^72^, ^73^, ^74^, ^75^, ^76^, ^77^, ^78^, ^79^, ^80^ ^]^

### 2.2 Technological and Biological Precedents for a Bio‐Information Processing Approach

It is important to emphasize that a fundamental assumption in using electrochemistry *as a tool for bio‐information processing* is that the molecular attributes responsible for the measured electrical/optical signals do not need to be known for a signature pattern to be valuable (i.e., the signals don't need to be mapped to chemistry). One familiar technological precedent is the electrocardiogram (EKG) which provides robust signatures of cardiovascular health without revealing molecular‐level details of the types and concentrations of the ions flowing across membranes. Similarly, electroencephalograms (EECs) have been used for a century to discern the stages and intensity of sleep, and these systems‐level measurements are guiding study of the underlying molecular and cellular mechanisms.^[^ ^81^, ^82^ ^]^ Finally, vital signs (e.g., body temperature) provide a real‐time measurement to assist in diagnosis and to serve as a target for treatment. In these examples, the measured signals emerge from molecular and cellular activities, yet the clinical value of these measurements is that their systems‐level pattern is robustly correlated to pathophysiological state (i.e., the pattern contains sufficient information to understand/act without mapping to the molecular/cellular activities occurring at lower length scales).

**Figure 5:**
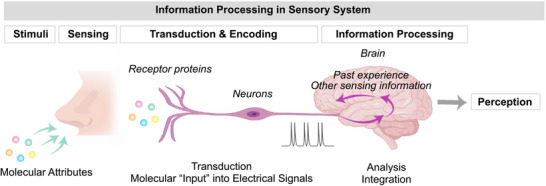
Biological sensory systems provide inspiration for an information processing approach. Sensory systems have evolved to efficiently extract actionable information from available stimuli: olfactory sensory neurons convert molecular attributes of an odor into electrical signals that are provided to the brain which couples multi‐modal sensory inputs with past knowledge to discern meaning.

The sensory systems provide the biological precedent for information processing. For instance, **Figure** **5**. shows that olfaction uses neurons to detect molecular attributes of an odor, and convert this information into electrical signals that are passed to the brain for: decoding; fusion with information from other sensory‐modalities; and integration with past experiences.^[^ ^83^ ^]^ Importantly, biological sensory systems have evolved under extreme selective pressures (e.g., the predator‐prey arms race) and provide valuable insights of how to extract actionable information from available stimuli under severe constraints of size, speed, and energy efficiency. Conceptually, it has been convenient to consider a “division”‐of‐labor: the sensory organ generates meaningful signals from available stimuli; and the brain discerns the meaning from these signals to generate perceptions that guide actions (e.g., behaviors). There have been many efforts to mimic olfaction's ability to convert molecular attributes into more convenient electrical signals. Specifically, “electronic noses” have enlisted arrays of sensors to mimic olfaction's use of hundreds of sensory neurons and its combinatorial coding strategy,^[^ ^66^, ^84^, ^85^ ^]^ and more recently, there have been efforts to mimic active sensing strategies (i.e., sniffing) for odor localization and tracking.^[^ ^86^, ^87^ ^]^ Interestingly, sensory scientists focus more on the brain's downstream information‐processing of olfactory inputs to understand biological responses (e.g., behaviors).^[^ ^88^ ^]^ Potentially, a more effective way to mimic olfaction would be to conceive it as a dynamic learning platform (not a standardized measurement system) where the “labors” of measurement and interpretation are integrated (rather than divided).

## 3 Electrochemistry as a Tool for Bio‐Information Processing: Case Studies

### 3.1 Case Study #1. Using Multiple Mediators to Characterize Redox‐properties of Melanin

An important application of mediated electrochemical probing has been for characterizing the redox activities of materials (e.g., to detect reversible redox activities^[^ ^56^ ^]^ and distinguish redox from conducting properties^[^ ^89^ ^]^). Redox activity is common to: various phenolic materials found in nature (e.g., melanin,^[^ ^51^, ^90^ ^]^ humics^[^ ^91^, ^92^ ^]^ and dietary antioxidants^[^ ^93^ ^]^); biomimetic materials (e.g., synthetic melanins^[^ ^94^ ^]^ and polydopamine^[^ ^95^ ^]^); and materials fabricated to interact with biology.^[^ ^96^, ^97^ ^]^ Despite the importance of redox‐active materials, there are few simple methods available to characterize redox‐activity for insoluble and non‐conducting materials.

**Figure 6:**
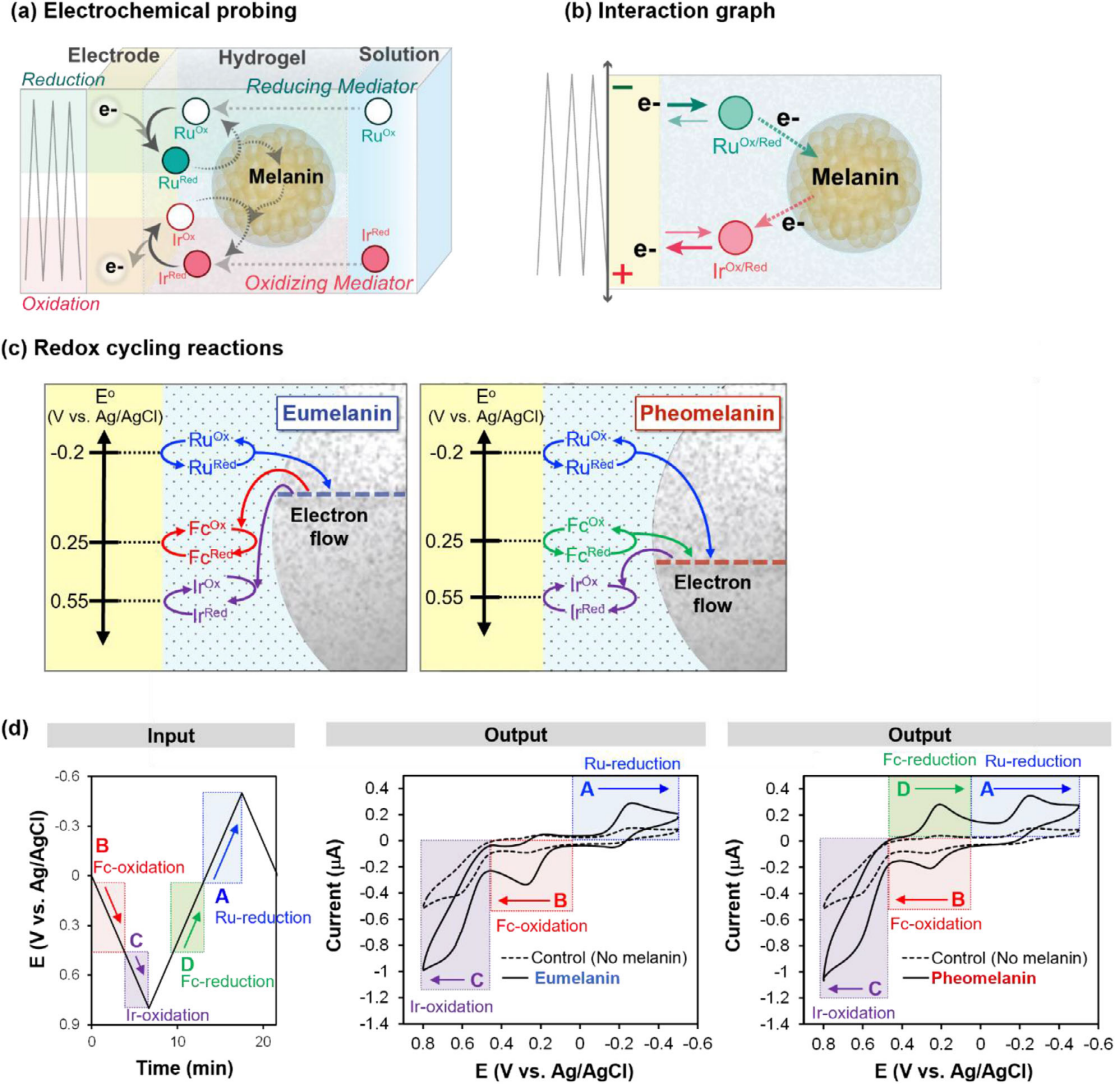
Discerning differences between eumelanin and pheomelanin by mediated electrochemical probing. a) Experimental approach in which melanins are entrapped within an inert hydrogel, and probed using mediators and an oscillating potential input. b) Interaction graph shows a network structure formed between 2 diffusible mediator “nodes” (Ru and Ir) and the immobilized melanin. c) Differences in redox‐cycling between the melanins and a third mediator, Fc, could reveal differences between eumelanin and pheomelanin. d) Experimental input curve and phase‐plane (*i‐E*) representations of the output electrical response. Reproduced under terms of the CC‐BY license.^[^ ^61^ ^]^ 2015, Springer Nature.

**Figure** **6**a illustrates mediated electrochemical probing for the case of melanins, which are ubiquitous biological pigments of poorly understood structures and functions. Some melanins (e.g., eumelanin) appear to confer beneficial photoprotective and antioxidant properties^[^ ^98^, ^99^, ^100^, ^101^ ^]^ while other melanins (e.g., pheomelanin) appear to have adverse photosensitizing^[^ ^102^ ^]^ and pro‐oxidant^[^ ^103^, ^104^, ^105^, ^106^ ^]^ activities. To explore differences between these two types of melanins, Figure 6a shows that we entrapped these insoluble melanins within a hydrogel at an electrode surface and then probed them using mediators.^[^ ^61^ ^]^ Each mediator can reversibly exchange electrons with the electrode, with the direction of electron exchange controlled by the imposed electrode potential. Also, each mediator can diffuse into the hydrogel to contact the entrapped melanin and exchange electrons in a thermodynamically‐controlled manner.

The interaction graph in Figure 6b, shows an abstraction of this experimental system as a 3‐node redox‐network formed between two diffusible mediators and the insoluble melanin. The Ru‐node is the most reducing (i.e., with the most negative *E°*). When the electrode is poised to reducing potentials (*E*<*E°_Ru_*; note the inverted scale where negative is at the top), Ru^Ox^ accepts an electron from the electrode and is reduced to Ru^Red^ which can diffuse into the film and donate its electrons to melanin. Melanin is an insoluble node with an intermediate *E°*. Melanin's redox‐state can be changed by accepting an electron from Ru^Red^ (note: the melanin moieties that undergo redox‐state switching are not known). The Ir‐node is the most oxidizing (i.e., with positive *E°*). When the electrode is poised to oxidizing potentials (*E*>*E°_Ir_*), Ir^Red^ donates an electron to the electrode and is oxidized to Ir^Ox^ which can diffuse into the film and accept an electron from melanin to switch melanin's redox‐state. When the imposed electrode potential is cycled from reducing to oxidizing *E* values, then electrons can sequentially enter the network through the Ru‐node, be stored in the melanin‐node, and then removed from the network through the Ir‐node. While both eumelanin and pheomelanin respond similarly to the Ru and Ir mediators, they respond differently to a third mediator, Fc, that has an intermediate *E°*. The response characteristics of these melanins support the model in Figure 6c that indicates pheomelanin has molecular moieties that possess a more oxidizing redox potential (compared to eumelanin).^[^ ^61^ ^]^

While several experimental approaches were used to support the above model, Figure 6d shows results from a single cyclic voltammetry (CV) approach in which synthetic versions of the two melanins were individually probed in the presence of the three mediators.^[^ ^61^ ^]^ Under the conditions used, these mediators are believed to reversibly transition between two redox states (oxidized and reduced) and, before probing, the bulk solution states were set as Ru^Ox^, Fc^Red,^ and Ir^Red^. Experimentally, the imposed input potential (i.e., voltage) was cycled over a relatively large voltage range (between +0.8 and −0.5 V vs Ag/AgCl) that spans the *E°* values of the individual mediators. As illustrated by the potential input curve in Figure 6d, cycling the voltage into the oxidative region passes through region “B”, where the Fc‐mediator is oxidized, and then to region “C”, where the Ir‐mediator is also oxidized. Upon reversing the input potential, the electrical signal in the initial region (not labeled in Figure 6d) is difficult to interpret because the locally‐generated Ir^Ox^ can be re‐reduced while Fc^Red^ can continue diffusing to the electrode to be oxidized (both oxidation and reduction reactions are occurring). Further cycling toward reducing potentials passes through region “D”, where the locally generated Fc^Ox^ is re‐reduced, and then region “A”, where the Ru^Ox^ mediator is reduced.^[^ ^61^ ^]^

The electrical responses to the imposed potential inputs are shown as current‐potential (*i‐E*) phase‐plane plots shown at the right in Figure 6d. The “Control” film that lacks melanin has no redox‐activity, and thus the dotted output curves of Figure 6d shows the response of the “Control” film to probing by the 3‐mediator solution and the oscillating input potential. The important point for the “Control” is that the response currents are relatively small over the range of imposed‐voltages. In contrast, the response currents for films containing the melanins are considerably amplified (relative to the Control). From a chemistry perspective, the amplification of mediator currents is characteristic of redox‐cycling reactions. Specifically, Ir^Ox^‐generated at the electrode diffuses into the film, where it can be re‐reduced by accepting electrons from the entrapped melanin, and then the Ir^Red^ can diffuse back to the electrode where it is re‐oxidized. Similarly, the electrochemically‐generated Ru^Red^ can diffuse from the electrode into the film, where it can donate an electron to the entrapped melanin and then diffuse back to the electrode, where it is re‐reduced.^[^ ^61^ ^]^

In comparison, the output response currents for the two melanins are similar in regions “C” (Ir‐oxidation) and “A” (Ru‐reduction), but they differ in regions “B” and “D” associated with the oxidation and reduction of the Fc‐mediator. Specifically, a prominent reduction peak in region “D” is observed for the pheomelanin (but not eumelanin) sample. This observation is the evidence that pheomelanin has molecular moieties with redox potentials that are more oxidative than eumelanin.^[^ ^61^ ^]^

The important points from Case Study #1 are: i) multiple mediators and an oscillating input potential sequence were used to probe melanins and generate high‐content output signals; ii) phase‐plane plots (i.e., *i‐E*) provide a convenient way to visualize differences between the two melanin‐containing films and a redox‐inactive Control film; iii) chemical intuition was used to interpret the response characteristics in terms or molecular mechanisms (i.e., redox‐cycling amplifies mediator currents); and (iv) the important function‐based conclusion (that pheomelanin has a more oxidative redox potential)^[^ ^61^ ^]^ is consistent with biosynthetic and chemical differences observed between pheomelanin and eumelanin.^[^ ^107^ ^]^

### 3.2 Case Study #2. Hypothesis‐Testing for a Proposed Mechanism for Clinical Intervention

**Figure 7:**
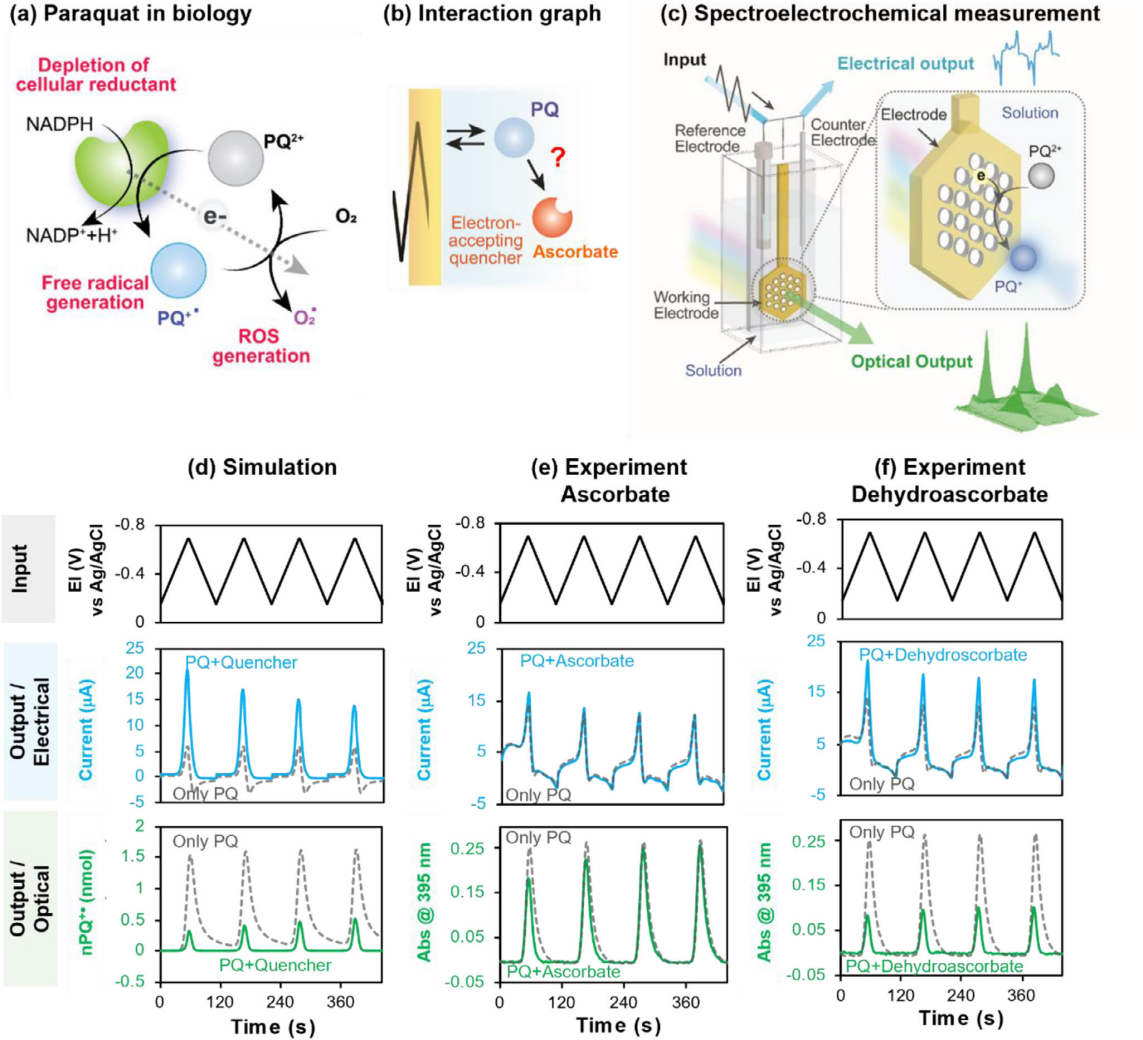
Electrochemistry can provide a simple tool to investigate biologically‐relevant redox mechanisms. a) The agricultural chemical paraquat is believed to be toxic through redox‐based mechanisms. b) An interaction graph for a proposed intervention for severe PQ‐poisoning: ascorbate (vitamin C) is proposed to scavenge the toxic PQ^+•^‐radical. c) Spectroelectrochemical approach to test the PQ^+•^‐scavenging hypothesis. d) Simulations from a minimal deterministic model suggest PQ^+•^‐quenching should result in amplified electrical signals and attenuated molecular signals (i.e., these molecular signals can be measured by spectroscopy). e) Experiments suggest PQ^+•^ is not quenched by Asc (electrical signals were not amplified and optical signals were not attenuated). f) Experiments suggest PQ^+•^ can be quenched by the oxidized dehydroascorbate. Adapted under terms of the CC‐BY‐NC‐ND license.^[^ ^52^ ^]^ 2023, Elsevier.

Electrochemistry has sometimes been used as an experimental tool to reveal detailed redox mechanisms in bioorganic chemistry (e.g., the oxidative metabolism of drugs^[^ ^108^, ^109^ ^]^ and interactions of reactive species^[^ ^110^, ^111^ ^]^). Experimentally, an electrochemical system can directly and quantitatively induce oxidation/reduction or generate reactive species (e.g., H_2_O_2,_^[^ ^112^ ^]^ NO^•^,^[^ ^113^, ^114^, ^115^, ^116^, ^117^ ^]^ O_2_^•−^ and HOCl) without the need for adding reagents or biological components that would complicate measurement and data interpretation.^[^ ^116^, ^117^ ^]^ In Case Study #2, we use electrochemistry to isolate and study a specific redox interaction associated with a proposed clinical intervention. Specifically, we studied the agricultural chemical paraquat (PQ), which is believed to be toxic through redox mechanisms. Specifically, **Figure** **7**a shows that the oxidized PQ^2+^ can accept an electron from cellular reductants, thus potentially disrupting cellular redox homeostasis. The product of this 1‐electron transfer reaction is the PQ^+•^ free radial which may be toxic. Finally, the PQ^+•^‐radical can donate its electron to O_2_ to generate toxic reactive oxygen species.^[^ ^118^, ^119^, ^120^, ^121^ ^]^ For cases of severe PQ‐poisoning, it has been suggested that ascorbate (Asc; vitamin C) could be used as a therapeutic intervention, and there has been some clinical evidence in support of this intervention.^[^ ^118^, ^122^, ^123^, ^124^, ^125^ ^]^ In theory, however, it should not be possible for the PQ^+•^‐radical to be directly quenched by donating an electron to ascorbate because they are both reductants.

In Case Study #2, we tested the hypothesis for quenching of the PQ^+•^‐radical as illustrated by the interaction graph in Figure 7b.^[^ ^52^ ^]^ Experimentally, we prepared solutions containing PQ with and without a putative quencher (e.g., Asc) and imposed oscillating electrode potentials near PQ's redox potential (*E°* = −0.64 V vs an Ag/AgCl). These oscillating electrical inputs enabled us to repeatedly switch this PQ molecule between its oxidized (PQ^2+^) and reduced (PQ^+•^) states while we measured the output responses to make inferences of the putative quenching interactions. Thus, the first generalizable aspect of this study is that electrochemistry sometimes allows biologically‐relevant redox interactions to be isolated and studied in the absence of confounding biological components and mechanisms.

The second generalizable concept is that often electrochemistry can be coupled with spectroscopy to increase the measurement dimensionality.^[^ ^52^ ^]^ Experimentally, Figure 7c shows that we used a honeycomb electrode that allows the simultaneous measurement of electrical and optical signals. Such spectroelectrochemical measurements can be especially informative because oftentimes, the spectral properties of a molecule vary with its redox‐state. For the case of PQ, the reduced PQ^+•^‐radical strongly absorbs UV light at 395 nm, while the oxidized PQ^2+^ has little UV‐absorbance at this wavelength. Thus, spectroelectrochemistry enables the simultaneous observation of electrical activities (associated with electron transfer) and molecular changes (associated with redox‐state switching).

The third generalizable concept in Case Study #2 is the use of a minimal deterministic model that reflects the relevant physical‐chemical mechanisms (i.e., diffusion and reactions) that can reveal the intrinsic structure (e.g., features) of the data.^[^ ^52^ ^]^ Compared to data‐driven machine learning (ML) and artificial intelligence (AI), the minimal modeling approach can make semi‐quantitative predictions with little or no training. Further, the minimal modeling approach is much simpler than a full theory‐based approach to create a digital twin. For our minimal modeling, we use a commercially‐available finite element reaction‐diffusion modeling package (electroanalysis interface of COMSOL)^[^ ^112^, ^116^ ^]^ with minimal effort to estimate parameters or account for details irrelevant to identifying discriminating features of the data (e.g., we ignore details such as the electrode shape and surface area).

The minimal model was used to simulate how a PQ solution or a mixture of PQ plus a quencher would respond to an oscillating imposed potential that could repeatedly reduce and oxidize PQ.^[^ ^52^ ^]^ As shown in Figure 7d the oscillating potential (upper plot) is predicted to induce: oscillations in currents associated with the repeated reduction and oxidation of PQ (middle plot); and oscillations in the appearance and disappearance of the PQ^+•^‐radical (bottom plot). The dotted curves in Figure 7d show predicted responses for the solution containing only PQ, while the colored lines show predictions for solutions containing PQ and a quencher (note the quencher is assumed to exchange electrons with PQ^+•^ but not exchange electrons with the electrode: Asc is not oxidized in this potential region). The important a priori prediction from this minimal model is that a quenching interaction is expected to amplify currents (due to redox‐cycling) but to attenuate the molecular signal (the levels of the PQ^+•^‐radical) due to quenching.

Experimentally, solutions were prepared containing PQ^2+^ (0.2 mM) or a mixture of PQ^2+^ (0.2 mM) plus ascorbate (0.5 mM) and tested using the spectroelectrochemical cell in Figure 7c.^[^ ^52^ ^]^ The oscillating input potential (top plot in Figure 7e) induced oscillations in the electrical response (current; middle plot) and spectral response (Abs@395 associated with the PQ^+•^‐radical; bottom plot). The simulated PQ^+•^‐levels at the bottom in Figure 7d are a proxy for the absorbance measurements shown at the bottom of Figure 7e. The first experimental observation in Figure 7e is that the initial oscillating current response is larger in the first (vs subsequent) cycle after which the oscillating responses appears to be steady (i.e., time‐invarying). In many cases, the steady response (vs an initial transient) contains the relevant information of systems‐behavior for our analysis. The second observation is that both the electrical and optical responses were similar between the two solutions (PQ and PQ+Asc). The absence of a current amplification and optical attenuation supports the conclusion that Asc cannot quench the PQ^+•^‐radical.^[^ ^52^ ^]^ This conclusion is consistent with the belief that ascorbate cannot accept an electron to quench the PQ^+•^‐radical.

In a separate experiment, we examined whether the oxidized dehydroascorbate could quench the PQ^+•^‐radical.^[^ ^52^ ^]^ Using similar experimental approach, Figure 7f shows that solutions containing both PQ and dehydroascorbate have amplified currents and attenuated optical absorbance consistent with dehydroascorbate serving to quench the PQ^+•^‐radical. Since ascorbate is readily oxidized to dehydroasorbate through various physiological mechanisms, it is possible that the administration of ascorbate may have benefits for cases of severe PQ‐poisoning through an indirect mechanism in which ascorbate is first oxidized (through uncharacterized physiological mechanisms) to generate a dehydroasorbate product that is capable of quenching the PQ^+•^‐radical.

In summary, Case Study #2 illustrates that electrochemistry may provide a simple approach to isolate redox interactions. Further, the coupling of electrochemical with orthogonal optical measurements allows the “fusion” of kinetic measurements of reaction rates (e.g., currents) with spectral measurements of molecular structure. Finally, a minimal deterministic model can make a priori predictions to guide data interpretation.

### 3.3 Case Study #3. Targeting Cysteine Residues: Mapping to Attributes of Protein Structure

**Figure 8:**
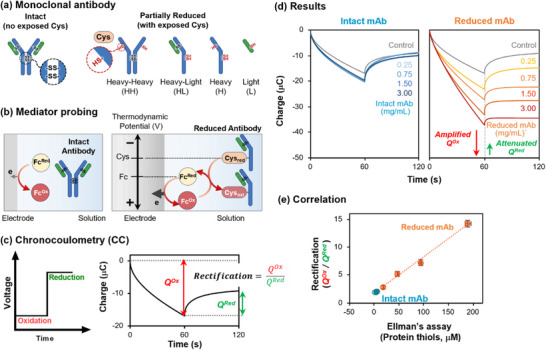
Converting attributes of protein structure into discriminating electrical signals. a) Intact and improperly folded, partially‐reduced mAb variants formed during biomanufacturing. b) Probing with Fc can detect mediator accessible redox activity (MARA) associated with accessible Cys residues. c) Step potential inputs in the presence of the Fc‐mediator can detect accessible Cys residues through redox‐cycling that amplifies oxidative charge (*Q^Ox^*) and attenuates reductive charge (*Q^Red^*) leading to rectification (*Q^Ox^/Q^Red^* > 1) of the electrical output signal. d) Experimental results with Intact and Partially‐Reduced mAb‐variants. (e) Correlation between electrical signal features (rectification; *Q^Ox^/Q^Red^*) and molecular attributes (protein thiols as measured by a standard Ellman's assay). Adapted under terms of the CC‐BY‐NC‐ND license.^[^ ^59^ ^]^ 2025, John Wiley and Sons.

Case Study #3 was motivated by the need for process analytic technologies (PATs) in biomanufacturing that can rapidly (in near real‐time) discern important protein attributes for process monitoring and control. Various protein‐based therapeutics are coming off‐patent providing an opportunity for the manufacture of less expensive generic alternatives. However, establishing structural equivalency of a protein‐based generic drug‐candidate is challenging because a protein's biological function depends on more than primary amino acid sequence but can also depend on folding (secondary and tertiary structure), associations (quaternary structure) and post‐translational modifications.^[^ ^126^, ^127^, ^128^ ^]^ To ensure efficient biomanufacturing, it is desirable that methods are available for rapid, near‐real‐time PATs that can discern when process conditions are leading to improper protein folding. Recently, the US National Institute of Standards and Technology (NIST) released a monoclonal antibody standard (NISTmAb) to assist biomanufacturers in their development of robust PATs to rapidly discern mAb attributes (e.g., to rapidly detect structural “fingerprints”). Through collaboration with both NIST and the US Food and Drug Administration (FDA), we investigated mediator‐probing as a means to rapidly discern intact NISTmAb from partially‐reduced and improperly‐folded variants that are generated during manufacturing.^[^ ^57^, ^59^, ^129^ ^]^ Specifically, in an intact mAb, the cysteine (Cys) residues form disulfide bonds that lead to the mAb's distinct quaternary structure involving heavy (H) and light (L) polypeptide chains. However, during bioprocessing, improper disulfide bond formation can lead to partially‐reduced mAb variants with exposed Cys residues, as illustrated in **Figure** **8**a.

To detect such exposed Cys residues, Figure 8b shows that we probed mAb‐containing solutions with a ferrocene dimethanol (Fc) mediator. As illustrated, when an oxidative potential is imposed, the reduced mediator (Fc^Red^) is oxidized at the electrode to form the oxidized mediator (Fc^Ox^), which then diffuses into the sample solution to probe for mediator‐accessible redox activity (MARA) associated with exposed Cys residues. Importantly, the Fc^Ox^ mediator is a weak oxidant – while it can oxidize Cys resides, it is unable to oxidize other amino acid residues (i.e., the Fc^Ox^ mediator selectively oxidizes Cys residues).^[^ ^64^ ^]^ If the sample contains accessible Cys residues, then the Fc^Ox^ can undergo redox‐cycling (Fc^Ox^ accepts electrons from Cys to regenerate Fc^Red^ which then can be re‐oxidized at the electrode). This redox‐cycling is expected to amplify the measured oxidative signals (*i^Ox^* or *Q^Ox^* = ∫*i^Ox^* d*t*) and attenuate reductive signals (*i^Red^* or *Q^Red^*).

In a chronocoulometric (CC) experiment, Figure 8c shows that we imposed a 60‐sec step change in oxidative potential (i.e., voltage) to solutions containing varying concentrations of either Reduced or Intact mAb, and then switched to a constant reductive potential. The measured output signals were quantified in terms of charges (*Q^Ox^* or *Q^Red^*; the cumulative electron transfer in each step). The CC measurements in Figure 8d show: i) the intact mAb (left plot) shows minimal differences in output *Q*‐curves with increasing concentrations which is consistent with the absence of surface exposed Cys residues for the intact mAb; and ii) the partially‐reduced mAb variant (right plot) shows increases in oxidation (i.e., amplification of *Q^Ox^*) and decreases in reduction (i.e., attenuation of *Q^Red^*) with concentration which is consistent with increasing levels of exposed Cys residues for the partially reduced mAb variant. We use these quantified electrical signal metrics (e.g., *Q^Ox^*, *Q^Red^* and rectification (*Q^Ox^*/*Q^Red^*))^[^ ^57^, ^59^ ^]^ as measurable features to discern intact from partially‐reduced mAb variants. This example is relatively simple and it was possible to map mediated electrochemical measurements to the relevant protein attributes (accessible Cys residues) through a single rectification feature‐metric (*Q^Ox^*/*Q^Red^*). Specifically, Figure 8e shows a linear correlation between this rectification metric and independent Ellman assays of the thiol content. (Note: since the intact mAb samples have no exposed Cys residues the data for these samples cluster near the origin in this plot.)^[^ ^59^ ^]^

In summary, we use the Fc mediator to transduce molecular attributes of protein structure (e.g., accessible Cys thiols) into interpretable electrical signals. From a chemistry perspective, the Fc mediator selectively oxidizes Cys residues because Fc's redox potential (*E°*) makes it a weak oxidant that is unable to oxidize other amino acid residues. From a PAT‐perspective, this Fc‐based mediator probing exploits the strengths of electrochemistry for a near‐real‐time measurement of signal features that correlate to important attributes of protein structure. Specifically, the detection of accessible Cys residues for the NISTmAb (and potentially proteins in general) provides insights into higher‐order tertiary and quaternary structural attributes associated with protein misfolding and/or unfolding.

### 3.4 Case Study #4. Probing for Proline Residues by Direct‐electrochemiluminescence (ECL)

**Figure 9:**
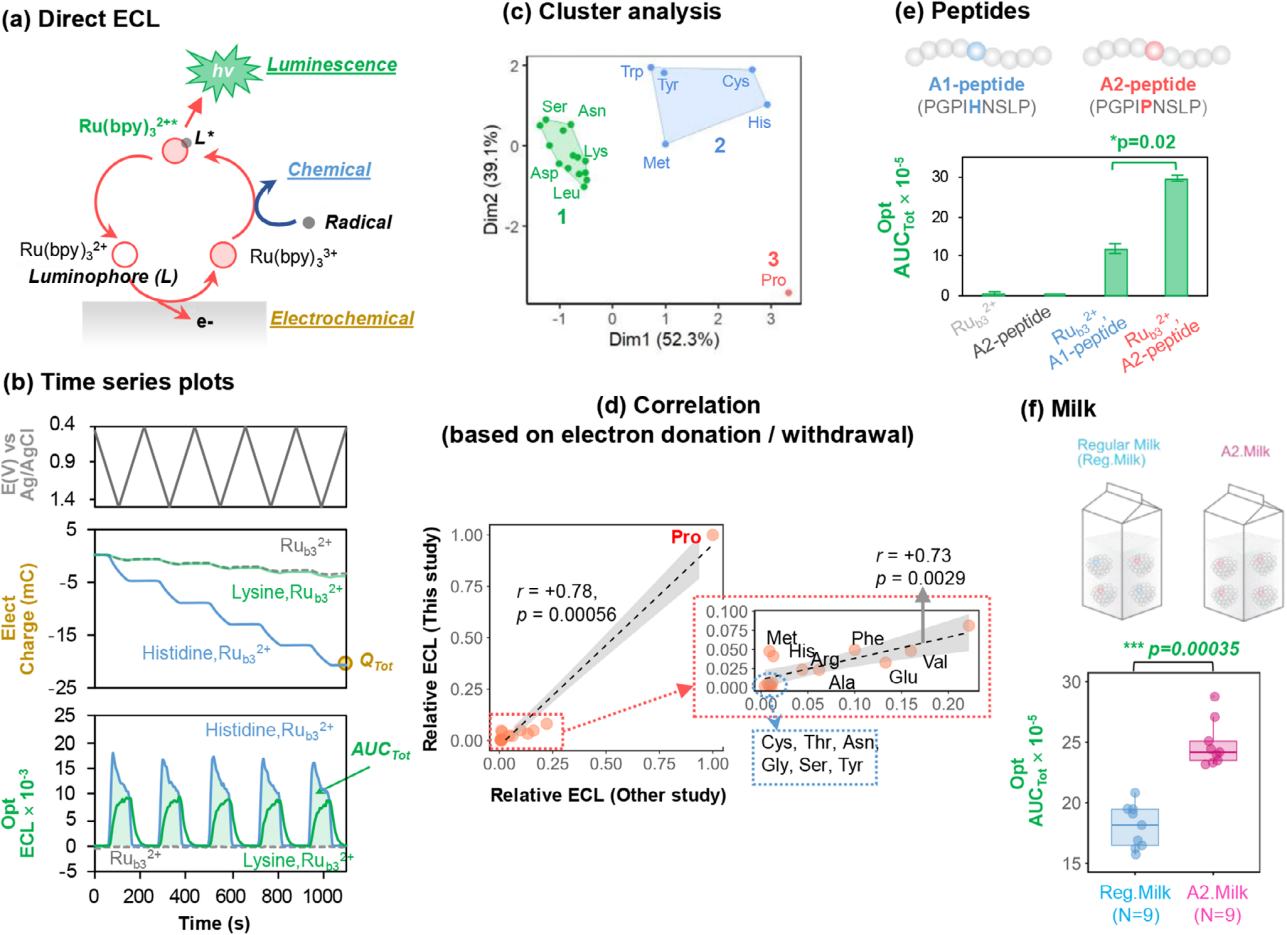
Direct electrochemiluminescence (ECL) to discern a single point mutation in β‐casein protein. a) Illustration that direct‐ECL can generate both electrical and optical signals. b) Experiment in which 5‐cycle cyclic potential inputs are imposed, and output response signals are quantified as total charge transfer (*Q_Tot_*) and Total luminescence (*AUC_Tot_*). c) Cluster analysis shows responses for the individual amino acids cluster into 3 groups based on their electrical and luminescence responses. d) The responses of single amino acids correlate to common physical‐organic chemistry parameters (electron‐donation/withdrawal). e) ECL‐discrimination of 9‐amino acid A1 and A2 peptide with a single amino acid substitution. f) ECL‐discrimination of A1 and A2 milks. Reproduced under terms of the CC‐BY license,^[^ ^65^ ^]^ 2024, John Wiley and Sons.

In Case Study #4, we used direct‐electrochemiluminescence (ECL) measurements in a complex matrix to map optical and electrical measurements to molecular attributes.^[^ ^65^ ^]^ **Figure** **9**a shows that a luminophore (Ru(bpy)_3_^2+^) is electrochemically oxidized to Ru(bpy)_3_^3+^, the oxidized Ru(bpy)_3_^3+^ diffuses into the solution where it can undergo chemical reactions with various components and, in some cases, these chemical interactions result in a luminescence signal (Note: ECL's molecular mechanisms remain incompletely understood).^[^ ^130^ ^]^ A common use for ECL is in immunoanalysis: antibody‐based molecular recognition is coupled with ECL as a sensitive signal transduction mechanism.^[^ ^131^, ^132^, ^133^ ^]^ In these cases, intermediate washing steps are typically used to remove potential interfering species from the assay to ensure the measured signal reflects antigen‐antibody binding. Here, we consider the use of direct‐ECL in which a complex sample is directly probed (i.e., without removing potential interferents).

The specific example for this study is the detection of a single amino acid variant involving the Pro‐to‐His substitution at position 67 in the β‐casein milk protein. Of the 209 amino acids in this protein, 35 are prolyl residues for the A2 variant and 34 for the A1 variant. This single amino acid substitution significantly affects casein's structure and digestion: the His67‐containing A1 variant (but not the Pro67‐containing A2 variant) is digested in the gastrointestinal tract to generate β casomorphin‐7 which is a bioactive opioid peptide reported to result in adverse responses in the gastrointestinal, cardiovascular, neurological and endocrine systems.^[^ ^134^, ^135^, ^136^, ^137^, ^138^ ^]^ Some consider A2 milk to be a healthier alternative to A1 milk, and thus various advanced analytical methods to differentiate A1 from A2 variants have been developed.^[^ ^139^, ^140^ ^]^

Initially, we examined the ability of direct‐ECL to distinguish individual amino acids. Figure 9b shows results from 5‐cycle time‐series cyclic voltammograms (CVs) of solutions containing either Lys or His. As illustrated, the output responses were quantified by two features (i.e., metrics): the total electrical charge transferred (*Q_Tot_*); and total area under the luminescence curve (*AUC_Tot_*). The cluster analysis in Figure 9c shows the 20 amino acids can be classified into 3 groups based on these electrical and optical metrics (most notably, Pro has an order‐of‐magnitude higher *AUC_Tot_*). The responses of the individual amino acids in Figure 9d show that our results agree with previous ECL results that correlated response to the amino acid's electron donation and withdrawal characteristics.^[^ ^141^ ^]^ Next, we used the same experimental approach (5‐cycle CVs), and showed that ECL can distinguish a 9‐amino acid peptide from A1‐ and A2‐ β‐casein despite the fact that both peptides are rich in Pro residues (3 vs 4; Figure 9e). Finally, we showed that ECL can discern the single amino acid variation of the casein proteins in the complex milk background. Specifically, the box plots in Figure 9f show that the luminescence (*AUC_Tot_*) is 1.3‐fold higher for the store‐bought A2 compared to the regular milks.^[^ ^65^ ^]^ It should be noted that one of the advantages of A2 milk as our experimental model is that molecular “tools” (e.g., purified A1 and A2 protein standards and selective antibodies) are commercially available, which allowed us to confirm that our direct‐ECL method is detecting differences associated with the A1 and A2 β‐casein proteins.^[^ ^65^ ^]^

In summary, Case Studies #3 and #4 show how a judicious selection of mediators allows important attributes of protein structure to be revealed. Unlike Case Study #3, the direct‐ECL measurement in Case Study #4 is not well understood, and thus the ability to discern a single Pro point mutation in a complex medium (i.e., milk) reflects more of a discovery than a design. In contrast to Case Study #3, where insights of molecular mechanisms (e.g., Fc‐redox‐cycling) provided a “theory” to select appropriate signal features (e.g., signal rectification *Q^Ox^/Q^Red^*), the limited understanding of direct‐ECL offers no such opportunity, and analysis relied on statistical correlations (i.e., data‐driven analysis). Potentially, direct‐ECL offers additional possibilities for transducing molecular information into readily measurable electronic (optical and electrical) signals. From a protein chemistry perspective, the sensitive detection of Pro residues is important because Pro's unusual secondary amine structure disrupts protein secondary structure and also limits proteolytic digestion.^[^ ^142^, ^143^ ^]^

### 3.5 Case Study #5. Redox‐Probing for Signature Patterns of Stress: Mapping to Biology

Next, we consider the measurement of oxidative stress (OxSt) from serum samples. OxSt is increasingly linked to various diseases,^[^ ^144^, ^145^, ^146^, ^147^, ^148^ ^]^ however, the molecular mechanisms and relevant molecular signatures (i.e., biomarkers) of OxSt are incompletely understood. As suggested in Figure 2c, stressors are generally believed to act through the generation of reactive species (e.g., ROS) that oxidize various molecular and cellular components (e.g., Figure 3 illustrates oxidation of components in the extracellular redox interactome). A common approach to identify markers of OxSt focuses on bottom‐up chemical measurements of the interactome nodes (e.g., the reactive species or the physiological reductants in serum; Figure 4b), protective antioxidant enzymes or signaling molecules (e.g., stress hormones of inflammatory cytokines). However, despite considerable effort, there is no generally‐accepted molecular biomarker(s) that reliably quantifies OxSt.

We are exploring a mediator‐based approach to measure OxSt that is based on three key assumptions. First, it is assumed that the molecular attributes in the serum's redox interactome contain a historical record of OxSt, and this record is stored in the pattern and extent of its oxidation (e.g., including the post‐translational oxidative modification of the protein pool).^[^ ^149^ ^]^ This assumption is common to several methods in which an oxidant or free radical is purposefully added to a sample to quantify the sample's remaining reducing/scavenging activities,^[^ ^150^, ^151^, ^152^, ^153^ ^]^ with the resulting measurements being inversely related to OxSt.^[^ ^149^ ^]^ Second, we assume this historical record is reasonably stable over time such that serum samples can be collected, processed and stored without loss of information. In our studies, we routinely test this assumption by comparing replicate samples that were measured at different times.^[^ ^154^, ^155^ ^]^ Finally, we assume this measurement provides useful information. In contrast to molecular approaches, we are not attempting to read/translate the historical record of OxSt into a list of interactome components (e.g., we are not attempting to “reconstruct” the nodes of the redox interactome). Rather, we are attempting to probe a sample to generate high‐information‐content signals that can be mapped to clinical assessments of health and disease.

**Figure 10:**
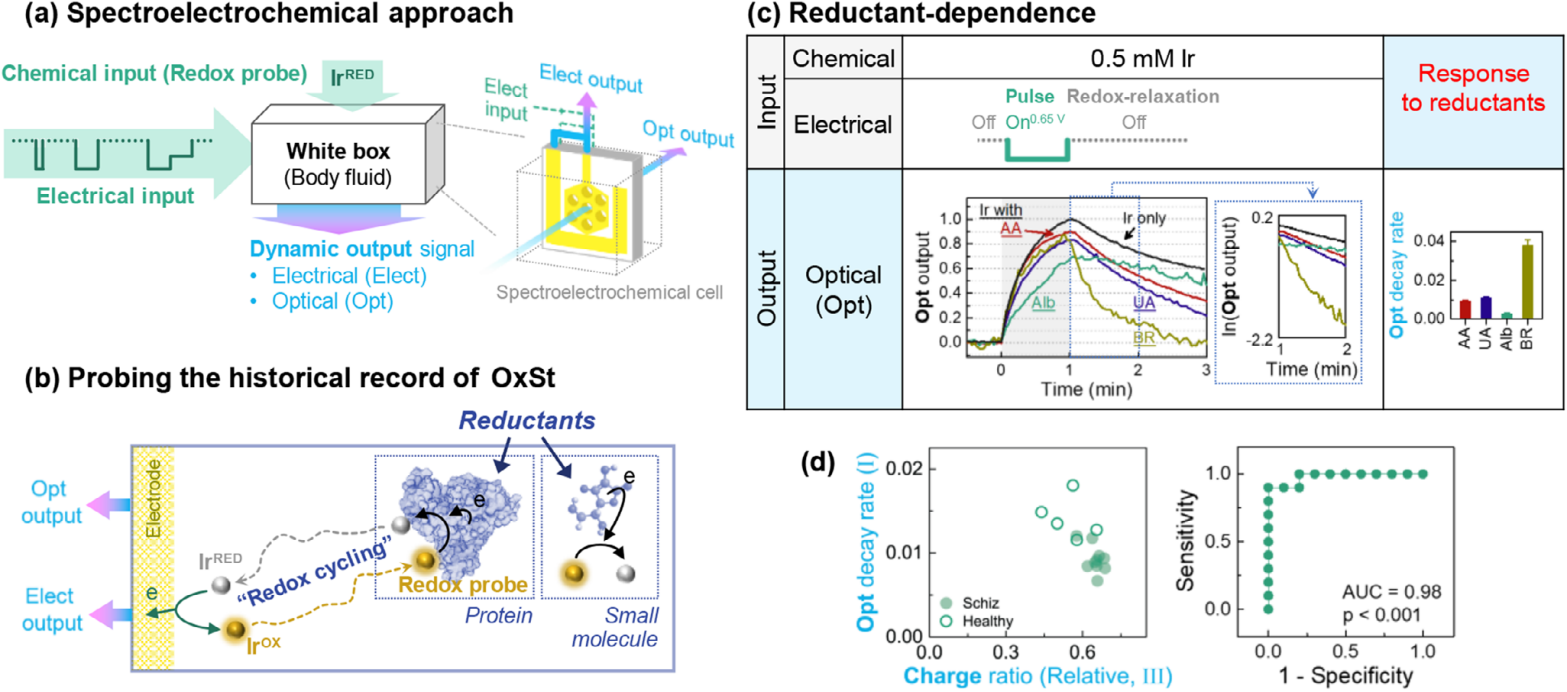
Spectroelectrochemical probing to discern signatures of oxidative stress (OxSt) from serum samples. a) Experimental approach in which a complex electrical input sequence is imposed, and output responses are measured electrically and optically. b) Ir‐probing aims to “read” the “historical record” of OxSt in a sample's interactome. c) Control experiments with individual serum components show the decay of Ir^Ox^’s optical signal varies with component: albumin (Alb); ascorbic acid (AA); uric acid (UA); and bilirubin (BR). d) Two signal metrics (i.e., features) could distinguish the schizophrenia and control groups based on their differing levels of oxidative stress. Reproduced with permission.^[^ ^63^ ^]^ 2018, Elsevier.

**Figure** **10**a illustrates one mediated spectroelectrochemical approach to obtain an objective measure of OxSt from serum samples.^[^ ^63^ ^]^ Our serum samples were collected in previous clinical studies and had been stored for months to years, and each sample is associated with an extensive set of clinical assessments (e.g., of patient symptoms at the time of sample collection). Each serum sample was diluted (10‐fold in phosphate‐buffered saline; PBS) and mixed with an iridium mediator (K_3_IrCl_6_; 0.5 mM final concentration) that is initially in its inactive, colorless reduced state (designated Ir^Red^). As illustrated in Figure 10b, probing is initiated by imposing an oxidative potential to electrochemically switch Ir^Red^ to its yellow‐colored oxidized state (Ir^Ox^). Ir^Ox^ is a relatively strong oxidant that can accept electrons from a wide range of reducible components (including various amino acid residues of proteins), and is especially sensitive to thiols.^[^ ^62^, ^156^, ^157^, ^158^ ^]^ The Ir^Ox^ that is generated at the electrode can diffuse into the sample where it can extract electrons from interactome components. The reduction of Ir^Ox^ by accepting electrons from interactome components can induce a redox‐cycling that amplifies the electrical signal and attenuates the yellow color (measured as absorbance at 488 nm) associated with Ir^Ox^. Figure 10a illustrates that we used simultaneous spectroelectrochemical measurements to read the historical record of OxSt and to translate this molecular‐based information into electronic (optical and electrical) signals.^[^ ^63^ ^]^

To illustrate the emergence and decay of an optical signal, we show results from a simple control study. Specifically, buffered solutions containing Ir^Red^ and a single component at a concentration expected for a 10‐fold diluted serum sample were probed by imposing a 1‐min oxidative input pulse (+0.65 V vs Ag/AgCl) and observing the absorbance at 488 nm which is characteristic of the yellow‐colored Ir^Ox^. As expected, Figure 10c shows that during the oxidative pulse, the optical signal increased for all solutions as Ir^Red^ is converted at the electrode into the yellow‐colored Ir^Ox^ that diffuses into the optical window of the honeycomb gold electrode. When the power was shut off and the generation of Ir^Ox^ ceased, Figure 10c shows the optical absorbance decreases both because the yellow‐colored Ir^Ox^ diffuses out of the optical window and the Ir^Ox^ is being re‐reduced by accepting electrons from the reducing components in solution. Importantly, the decay in optical absorbance varies depending on which reductant was present, and the decay rate presumably reflects the metabolite's intrinsic reactivity. Also important is that the decay in optical signal is readily quantifiable (e.g., by a first‐order rate constant) to yield a signal metric (i.e., feature) that contains chemically‐dependent information.

The specific clinical application for this study is schizophrenia, which is a serious mental health disorder that has been associated with OxSt.^[^ ^146^, ^147^, ^159^, ^160^, ^161^ ^]^ Thus, our goal was to discern whether Ir‐probing of serum could detect differences in responses between a schizophrenia (SCZ; N = 10) and healthy control (HC; N = 5) groups. From preliminary empirical studies, we decided to probe the samples using an input‐sequence of 3 potential *E*‐pulses; and to characterize the output responses in terms of 14 quantitative features (i.e., 5 electrical, 6 optical, and 3 cross‐modal). Statistical machine learning was used (e.g., Wilcoxon nonparametric test and the receiver operating characteristic (ROC) analysis) to analyze this data. [Note: the experimental and data analysis methods are further described in the original manuscript.]^[^ ^63^ ^]^ Figure 10d shows that two signal feature metrics (one optical and one electrical) showed statistically significant differences between the SCZ and HC groups. In addition, these two metrics could be combined using multiple logistic regression analysis to provide even greater discriminating capabilities between the SCZ and HC groups.

Case Study #5 illustrates the coupling of mediators with tailored *E*‐input sequences to generate information‐rich electronic output signals (optical and electrical). These output response signals were then characterized by a set of quantitative signal metrics (i.e., features) that serve as variables in data‐driven statistical analysis to reveal discriminating patterns in the data. This Case Study also illustrates the direct‐mapping of simple, rapid, and inexpensive electronic measurements to clinical assessments (e.g., a diagnosis of schizophrenia) without mapping through intermediate chemical analyses of the specific molecular attributes responsible for the measured electronic signals. We envision that such simple electronic measurements could provide a rapid and inexpensive approach to measure OxSt both for laboratory research and at the point‐of‐care (potentially as a vital sign). Further, we envision that such electronic measurements could provide systems‐level information to complement more‐traditional bottom‐up molecular approaches to characterize OxSt.^[^ ^62^, ^63^, ^154^, ^155^, ^162^, ^163^ ^]^ Thus, the longer‐term goal is to develop redox‐based electronic measurements that could be as reliable and clinically useful as more conventional electronic measurements of biology's ionic electrical modality (e.g., electrocardiograms and electroencephalograms).

### 3.6 Case Study #6. Redox‐based Electrogenetic Actuation of Gene Expression

**Figure 11:**
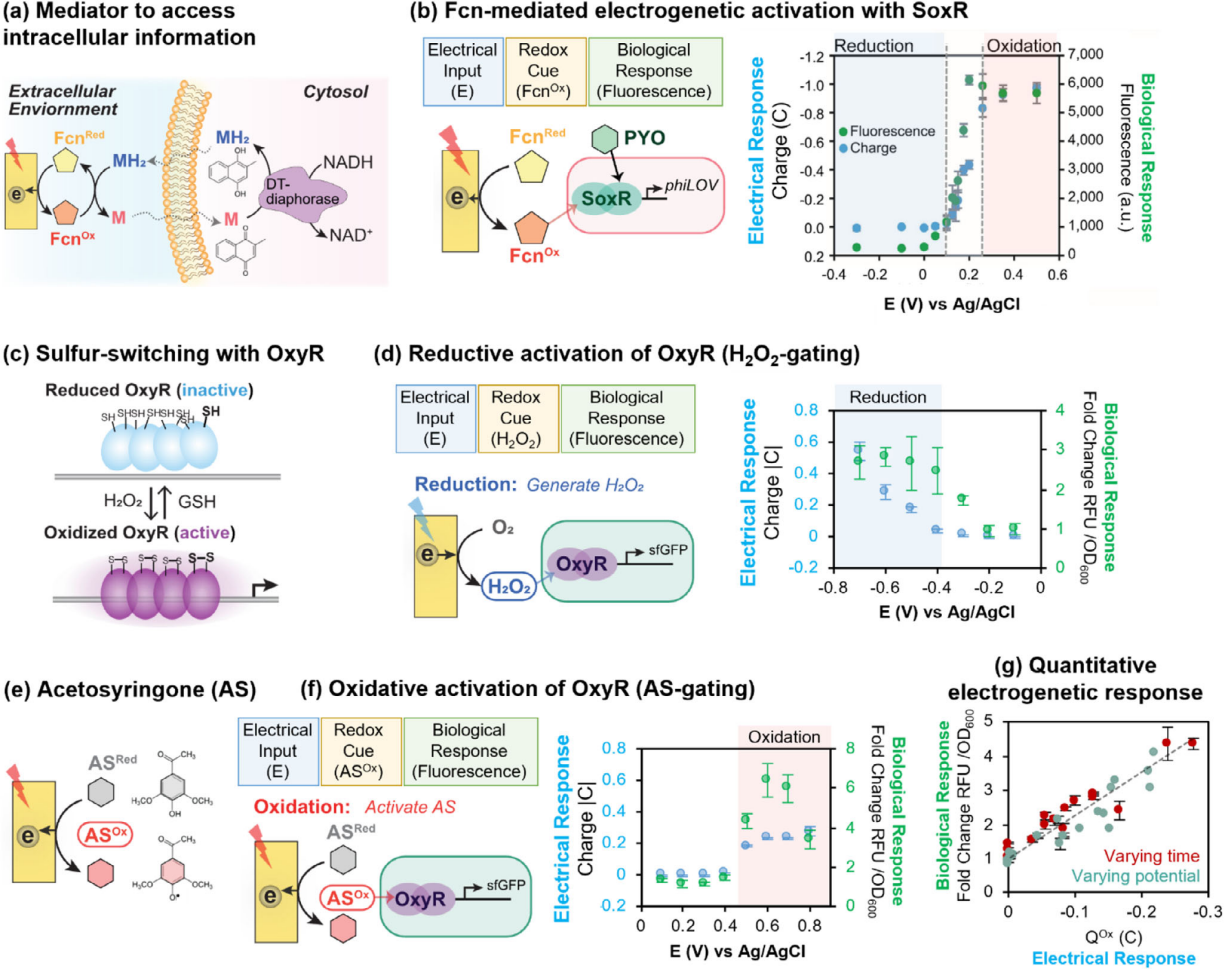
Electrogenetic actuation of gene expression. a) Mediator pairs (MH_2_/M and Fcn^Red^/Fcn^Ox^) allowed extracellular electrochemical measurement of intracellular activities. b) The Fcn mediator can induce gene expression from the *E. coli* redox responsive SoxRS operon: the phase‐plane plots (*Q^Ox^‐E* and *Fluorescence‐E*) show how the electrical input (*E*) is gated to yield the redox‐cue (Fcn^Ox^) that induces the electrical (*Q^Ox^*) and biological (*Fluorescence*) responses. Reproduced under terms of the CC‐BY license.^[^ ^55^ ^]^ 2017, Springer Nature. c) A second *E. coli* redox‐responsive regulon, OxyR, uses a sulfur‐switching mechanism. d) Electrochemically‐generated H_2_O_2_ serves as a redox‐cue to gate the electrical and biological responses of the OxyR regulon to imposed reducing potentials. e) The plant signaling molecule acetosyringone (AS) serves as a redox‐state‐dependent cue. f) Electrode‐oxidized AS serves as a redox‐cue to gate the electrical and biological responses of the OxyR regulon to imposed oxidizing potentials. g) The correlation between biological response (*Fluorescence*) and electrical response (*Q^Ox^*) to the imposed electrical input (*E*). Adapted under terms of the CC‐BY license.^[^ ^60^ ^]^ 2024, Springer Nature.

As noted, redox is a native biological modality important for bioenergetics, immune defense, and signaling, and this redox modality is also accessible to electronics through electrochemistry. Thus, redox provides a modality to “connect” biology to electronics.^[^ ^10^, ^164^, ^165^, ^166^, ^167^ ^]^ Such a redox connection was illustrated by an early study that demonstrated the use of two mediators to access intracellular redox information (**Figure** **11**a). One mediator (menadione/menadiol; M/MH_2_) is believed to enter the cell and acquire electrons from NADH (via the enzyme DT‐diaphorase) and possibly also from the mitochondrial electron transport chain. A second mediator (ferricyanide/ferrocyanide; Fcn^Ox^/Fcn^Red^) is believed to be unable to enter the cell but can accept electrons from MH_2_ that passes into (or through) the cell membrane, and this second mediator can shuttle electrons to the electrode.^[^ ^168^ ^]^ This study illustrates that mediators allow intracellular activities to be probed using extracellular electrochemical measurements.^[^ ^169^, ^170^, ^171^ ^]^ Importantly, mediators that can transfer electrons out of (or into) a cell offer the potential to alter the cellular redox balance and shift metabolic pathways toward the generation of more oxidized (or more reduced) products. Potentially, such redox‐based electro‐actuation could be used to direct electrobiosynthesis and optimize yields.^[^ ^172^, ^173^, ^174^ ^]^

In addition to using the redox modality to observe cellular activities (e.g., for sensing) and alter metabolic fluxes, it is also possible to use redox “cues” to actuate cells by opening ion channels for neuronal excitation,^[^ ^77^, ^112^, ^116^, ^117^, ^193^ ^]^ or by inducing gene expression through redox‐responsive regulons (**Table** **3**).^[^ ^175^, ^177^, ^178^, ^185^, ^187^, ^188^, ^189^, ^194^, ^195^, ^196^ ^]^ For instance, *E. coli* has two well‐known redox‐responsive regulons that are believed to regulate responses to reactive oxygen species and oxidative stress.^[^ ^197^ ^]^ The SoxRS regulon uses a transcription factor with an iron‐sulfur cluster that is responsive to superoxide,^[^ ^198^, ^199^, ^200^ ^]^ and Figure 11b illustrates that a re‐wiring of this regulon allows for redox‐based electrogenetic activation of gene expression (e.g., to produce a fluorescent protein). In initial studies, two mediators, Fcn^Red^ and the bacterial metabolite pyocyanin (PYO^Ox^), were used to drive gene expression. The plot in Figure 11b shows that at reducing input potentials (imposed *E*‐values more negative than Fcn's *E°*): no oxidative currents are drawn (no electrical response); Fcn^Red^ is not oxidized and therefore the redox cue Fcn^Ox^ is not generated; and no biological response is observed (no up‐regulation of fluorescent protein expression). In contrast, when the imposed potential is increased to become more oxidative (*E ≈ E°*), the phase‐plane plots in Figure 11b show monotonous increases in both the electrical response (*Q^Ox^‐E*) and the biological response (*Fluorescence ‐E*). The shape of these phase‐plane plots indicates that Fcn (*E°≈* +0.2 V vs Ag/AgCl) serves as a voltage‐gate that controls how the input electrical potential is converted into a redox cue responsible for the biological response.^[^ ^55^ ^]^

**Table 3:** Recent advances in redox‐based actuation of biological systems.

Redox‐responsive regulon	Cell and transcriptional regulation elements	Redox stimulus; environmental conditions	Working electrode	Modulated cellular activity	Refs.
**Bacterial**					
	***Non‐electroactive bacteria***				
SoxRS	*E. coli*; transcriptional regulator SoxR, promoter P_soxS_	Ferrocyanide (K_2_Fe(CN)_6_) electrochemical oxidation (> +0.3 V) in presence of pyocyanin; anaerobic	Gold wire electrode	Expression of phiLOV FP; CheZ‐modulated bacterial swimming; expression of AHL signal as cue for downstream cells to express phiLOV FP.	[55]
			Gold wire electrode	Tunable SoxRS‐based electrogenetic CRISPRa system (eCRISPRa) and transient silencing of target genes using dCas9 with spatiotemporal resolution.	[175]
			Gold‐coated glass wafer	Expression of phiLOV FP and study of cell electrogenetic activation mechanism (electrochemical vs chemical).	[176]
		Ferrocyanide (K_2_Fe(CN)_6_) electrochemical oxidation (> +0.3 V) in presence of pyocyanin; aerobic	Carbon paper disc electrode	Expression of sfGFP FP with a redox‐responsive promoter library.	[177]
		Ferrocyanide (K_2_Fe(CN)_6_) electrochemical oxidation (> +0.3 V) in presence of phenazine methosulfate; anaerobic	Platinum wire electrode	Electrogenetic cellular recorder for digital‐to‐biological data storage. Electronic actuation leads to an increase in copy number of a trigger plasmid (pTrig) due to activation of P1 replication system. A “writing module” incorporates this information via acquisition bias for pTrig‐derived spacers in expanding CRISPR arrays, demonstrating ability to encode binary data into CRISPR arrays of bacterial cells by electrical stimulation.	[178]
OxyRS	*E. coli*; transcriptional regulator OxyR, promoter P_oxyS_	H_2_O_2_ generation via electrochemical reduction (< ‐0.4 V); aerobic	Gold‐patterned silicon wafer chip, cells immobilized on chip with gold‐binding peptide	Expression of AHL signal as cue for downstream cells to (a) express β‐galactosidase, catalyzing PAP generation and detection via electrochemical oxidation for BioLAN connectivity, and (b) upregulate TolAIII‐mediated membrane porosity for release of GMCSF and DsRedExpressII FP.	[179]
			2 mm standard gold disc electrode	Expression of AHL signal as cue for downstream cells to express HPr in isogenic null mutants to increase growth rate and modulate population in a co‐culture.	[180]
			2 mm standard gold disc electrode	Expression of AHL signal as cue for downstream cells to (a) express *aroG, aroE, tyrA* for inducible tyrosine production, or (b) express HPr in cells constitutively expressing tyrosine to increase growth rate and population in co‐culture.	[181]
			2 mm standard gold disc electrode	Bacterial lysis by expression of phage lysis protein *lysisE* either (a) directly regulated by OxyR or (b) regulated by AHL signal – which is regulated by OxyR in a second cell population – with programmed time delay.	[182]
			ITO‐coated glass wafer with PEG‐SH/cell biofilm electrodeposited using ferrocene mediator	OxyRS‐based eCRISPR systems: (a) eCRISPRa in cells electrodeposited in an “artificial biofilm” modulated expression of AHL signal as cue for planktonic cells to express sfGFP FP, (b) eCRISPR inhibition of native AI‐2 signaling and “switch” to *P. aeruginosa*‐sourced AI‐1 signaling with eCRISPRa of *lasI*. Development of BioSpark system enabled automated dynamic control of gene expression.	[183]
		Acetosyringone (AS) electrochemical oxidation (> +0.5 V); aerobic	Gold wire electrode	Induction of a single promoter (OxyR‐regulated signaling of P_oxyS_) by two different mechanisms at distinct potential ranges: H_2_O_2_ generation at reducing potentials or AS oxidation at oxidizing potentials. Demonstrated dynamic programming of sfGFP FP expression with varying response dynamics based on potential input.	[60]
			ITO‐coated glass wafer with PEG‐SH/cell biofilm electrodeposited using ferrocene mediator	Expression of mScarlet FP by induction of a single promoter (OxyR‐regulated signaling of P_oxyS_) – by H_2_O_2_ generation at reducing potentials or AS oxidation at oxidizing potentials – in an electrodeposited ‘artificial biofilm.'	[59]
	***Electroactive bacteria***				
Arc	*Shewanella oneidensis*; sensor kinase ArcS & transcriptional activator ArcA, promoter P_nqr2_	Cathodic current (‐0.4 V); anaerobic	Graphite felt electrode	Expression of *lacZ* upon exposure to a cathodic current, but not an anodic current (+0.7 V). In situ time‐course monitoring of anaerobic FP evoglow‐Bs2 expression in electrode‐associated biofilms.	[184]
Arc	*Shewanella oneidensis*; sensor kinase ArcS & transcriptional activator ArcA, exogenous promoter P_feo_ from *E. coli*	Anodic current (+0.7 V); anaerobic	Graphite felt electrode	Expression of *lacZ* upon exposure to an anodic current, but not a cathodic current (‐0.4 V).	[184]
Rex	*Shewanella oneidensis*; exogenous transcriptional inhibitor Rex and promoter P_IP_ from *Bacillus subtilis*	Elevated NADH/NAD^+^ ratio upon switch to ‐0.6 V; anaerobic	Carbon felt electrode	Dual‐stage microbial electro‐fermentation with electro‐controlled regulation of isobutanol synthesis. In the first stage, application of +0.5 V triggered biofilm formation while repressing isobutanol synthesis genes due to low NADH/NAD^+^ ratio. In the second stage, application of ‐0.6 V increased NADH/NAD^+^ ratio, derepressing isobutanol synthesis genes.	[172]
**Mammalian**					
AlcR	CHO; transcriptional activator AlcR, promoter P_AIR_	Electrochemical oxidation of ethanol to acetaldehyde with DC application between 30–140 mA or AC of 50 Hz	Platinum anode and cathode	(a) Expression of human glycoprotein SEAP and measurement of SEAP production with an enzymatic‐optical process yielding an electronic output; development of a frequency modulation detector. (b) Expression of bone morphogenetic protein BMP‐2 to stimulate neonatal rat cardiomyocytes; development of a frequency generator.	[185]
NFAT	HEK and pancreatic β cells; transcriptional activator NFAT, promoter P_NFAT3_, voltage‐gated calcium channel Ca_V_1.2	Membrane depolarization by application of square unipolar +50 V pulses	Platinum anode and cathode	(a) Transcription‐based insulin production in HEK cells; (b) rapid insulin and luciferin release via electrostimulated vesicular secretion in pancreatic β cells. Electrogenetic cells expressing insulin were applied to Type 1 diabetic mice; bioelectronic implant enabled remote control of electrostimulation.	[186]
			Platinum‐coated cell chamber	Piezo‐triggered electro‐inducible vesicular secretion for insulin release.	[187]
NRF2/KEAP1	HEK; ROS sensor KEAP1, transcriptional activator NRF2, promoter P_DART_	Exposure to 4.5 V DC	Platinum anode and cathode or platinized acupuncture needles	Reversible expression of (a) SEAP or (b) insulin. Electrogenetic cells expressing insulin were applied to Type 1 diabetic mice, with battery‐powered device enabling remote control of electrostimulation.	[188]
			3D‐printed borophene honeycomb scaffold with borophene nanofibers	Expression of vascular endothelial growth factor VEGFA to promote vascularization. Electrostimulation of implant applied for wound‐healing in rats.	[189]
		Magnetoresponsive ROS‐generating multiferroic nanoparticles as receivers of an externally applied EMF	n/a (Helmholtz‐coil device for EMF generation)	Electromagnetically stimulated insulin expression; encapsulated electrogenetic cells were implanted in Type 1 diabetic mice subjected to magnetic field stimulation.	[190]
NF‐κB	Human cervix carcinoma Hep‐2c; nuclear factor NF‐κB, promoter P_NF‐κB_	Eight 100‐µs EF pulses with amplitude 0.165 kV/cm	n/a (square wave electric pulse electroporator for EF generation)	Expression of SEAP in response to microsecond‐duration pulsed electric fields.	[191]
	***Electrocatalysis***				
Nitric oxide (NO)‐mediated signaling pathways	HEK and primary rat hippocampi; transient receptor potential vanilloid family member 1 (TRPV1) ion channels, post‐transcriptional cleavage linker p2A under promoter CaMKIIα	Electrochemical NO generation by iron sulfide‐based nanocatalysts and application of −1.75 V	Platinum electrodes or Pt‐Fe_3_S_4_ nanoclusters deposited on gold‐coated tungsten microwires	Electrochemical NO generation drove Ca^2+^ influx into cells for expression of mCherry and GCaMP6s FPs and a broad neuronal human synapsin promoter. Observed increase in cGMP levels indicating activation of NO receptor guanylate cyclase (sGC). Implanted NO delivery probe enabled NO‐mediated stimulation in mice.	[117]
	HEK; TRPV1 ion channels, post‐transcriptional cleavage linker p2A under promoter CaMKIIα	Electrochemical NO generation from electrocatalyst FeCuMoS_4_ (−1.2 V)	Platinum anode and carbon electrode cathode loaded with FeCuMoS_4_	Electrochemical NO generation drove Ca^2+^ influx into cells for expression of mCherry and GCaMP6s FPs. Selectivity of NO or NH_3_ generation conferred by electrocatalyst.	[192]
Ammonia (NH_3_)‐mediated signaling pathways	HEK; otopetrin 1 (OTOP1) ion channels	Electrochemical NH_3_ generation from electrocatalyst Cu_2_MoS_4_ (−1.2 V)	Platinum anode and carbon electrode cathode loaded with Cu_2_MoS_4_	Electrochemical NH_3_ generation caused cell cytosol alkalinization, activating OTOP1 for expression of fluorescent pH indicator SEpHluorin. Selectivity of NO or NH_3_ generation conferred by electrocatalyst.	[192]

Abbreviations: FP, fluorophore; AHL, acylated homoserine lactone; PAP, *p*‐aminophenol; BioLAN, biological local area network; GMCSF, granulocyte macrophage colony‐stimulating factor; HPr, an *E. coli* phosphotransferase system protein; ITO, indium tin oxide; CHO, Chinese hamster ovarian cells; SEAP, secreted human alkaline phosphatase; HEK, human embryonic kidney cells; ROS, reactive oxygen species; EMF, electromagnetic field; EF, electric field.

A second redox‐responsive *E. coli* regulon involves the OxyR transcription factor that acts through a cysteine‐based sulfur‐switching mechanism as illustrated in Figure 11c.^[^ ^201^, ^202^, ^203^ ^]^ In nature, sulfur‐switching is believed to be cued by H_2_O_2_ which can also be electrochemically‐generated when an electrode is poised at a sufficiently reducing (i.e., negative) potential to reduce oxygen. As illustrated in Figure 11d, OxyR can be activated by imposing a reducing potential in the presence of O_2_ to generate the H_2_O_2_ redox cue.^[^ ^179^, ^180^, ^181^, ^204^ ^]^ The phase‐plane plot in Figure 11d shows when the electrode potential is poised at potentials that are too oxidative (*E* > −0.2 V vs Ag/AgCl), then there is: no electrical response (*Q^Red^* ≈0); no generation of the redox cue (H_2_O_2_); and no observed biological response (no fluorescent protein expression). When more reducing potentials are imposed (*E* < ‐0.3V), then both electrical (*Q^Red^*) and biological (*Fluorescence*) responses were observed, consistent with the generation of H_2_O_2_ redox‐cue. Again, the shape of the electrical and biological phase‐plane plots indicates that H_2_O_2_ serves as a reductive‐gate for expression from OxyR.

Subsequent studies showed that expression from OxyR can also be induced by the plant signaling molecule acetosyringone (AS) – but only if AS is oxidized.^[^ ^60^ ^]^ Figure 11e shows the putative oxidation reaction for AS and indicates that AS is inert in its normal, reduced, state, but is activated upon oxidation. (Note: it is hypothesized in some plant‐pathogen interaction mechanisms, that AS is oxidatively‐activated during an oxidative burst.^[^ ^205^, ^206^ ^]^) The schematic and phase‐plane plots in Figure 11f indicate that AS‐oxidation serves as an oxidative gate for expression from the OxyR promoter. Finally, Figure 11g shows that the biological response (*Fluorescence*) is correlated to the “intensity” of the electrical response (*Q^Ox^*) to the imposed electrical input (*E*). Overall, the results with OxyR demonstrate that gene expression can be induced electrochemically under either reducing conditions (sufficient for the electrochemical‐generation of H_2_O_2_) or oxidative conditions (sufficient for oxidation of AS), but not under intermediate input potentials.^[^ ^60^ ^]^

In our earlier Case Studies, we focused on accessing information (e.g., for sensing) and emphasized the conversion of molecular attributes into interpretable electronic signals. This emphasis is consistent with a molecular paradigm that biological information is coded in molecular identity and structure. Case Study # 6 illustrates the limits of this molecular‐structure paradigm when applied to redox biology. Two biological signaling molecules of distinctly different molecular structures (H_2_O_2_ and AS) can engage a thiol switching mechanism responsible for upregulation of the same OxyR regulon. Further, the activities of these signaling molecules are highly dependent on redox‐context: H_2_O_2_ is electrochemically‐generated under reducing potentials; AS is activated under oxidizing potentials; and neither redox‐signal is generated under intermediate potentials. Thus, characterizing *redox‐activities* may be as relevant as determining *molecular structure* for advancing our knowledge in redox‐biology. Since electrochemistry is well‐suited for characterizing redox‐activities, it could emerge as a critical tool in redox biology.

## 4 Perspective And Future Vision

This Perspective has two broad themes. First, redox is a native biological modality that is accessible to electronics through electrodes: thus, redox provides a modality for bi‐directional bio‐electronic communication for sensing and actuation. Importantly, the electron‐based redox modality is fundamentally different from biology's more familiar ion‐based electrical modality, and thus, the methods to establish bio‐electronic connectivity will also differ. Specifically, the ion‐based modality can be accessed through electric fields, while access to the redox modality generally requires electron‐transfer electrochemical reactions. Second, while electrochemistry has well‐known limitations for chemical analysis, it has significant strengths (simplicity, speed, sensitivity, and portability) for accessing redox information and converting it into interpretable electronic signals. Since we view the electrode as a tool for information processing (Figure 4c) and analogous to a sensory organ in biology (Figure 5), then we envision that the metrics for success will emerge more from considerations of the 5Vs for big data and less from the characteristic metrics for chemical analysis (i.e., sensitivity and selectivity).

The idea that electrodes can be redox‐based bio‐information‐processors is still emerging. We use a set of Case Studies to illustrate various unconventional electrochemical approaches to increase the information‐content of the evoked redox‐based signals: combining mediators; tailoring electrical input sequences; and measuring output responses through multiple modalities (electrical and optical). These Case Studies further illustrate theory‐guided (i.e., mechanism‐based) approaches to extract features from the output signals, and data‐driven machine learning to recognize patterns. We believe these Case Studies are a starting point that suggest broader opportunities. While optimistic, we acknowledge that there will likely be limitations to: how much redox‐based information from the molecular attributes can be “converted” into electronic signals; how much information can be compressed into a finite number of signal‐features; and how to balance information‐content against measurement‐speed. Yet, these limitations are not unique, and are shared by analytical chemical approaches that aim to distill omic measurements into a small subset of molecular biomarkers that can provide a clinician with timely and actionable information. Importantly, Case Study #6 provides a cautionary tale to imposing a molecular‐paradigm to redox biology: this Case Study indicates that activities and contexts, and not static structure, may be paramount to understanding and participating in the redox‐based flow of information.

In the future, we envision that the speed of electrochemical measurements could allow real‐time data analysis and feedback control to enable autonomous learning for: discovering unique signatures (e.g., of health and disease); resolving interactome network structures; or optimizing biomanufacturing processes. Further, the miniaturizability of electrode measurements should allow Moore's Law scaling for deployable devices in medicine, agriculture, environmental science, and consumer products (e.g., wearables). Finally, we envision that bi‐directional redox interactions could enable electronic access to information in complex biological environments (e.g., the gut and rhizosphere), and this is complementary to emerging efforts that aim to enlist electrochemistry for cleaner manufacturing (i.e., electrosynthesis) and energy‐harvesting (e.g., the generation of electricity from solar or chemical energy sources). In summary, redox's ability to span biology and electronics could enable an integration of the internet‐of‐things with the web‐of‐life.

## Conflict of Interest

The authors declare no conflict of interest.

## Supporting information



Supporting Information
