# Supplementary material for: Electrochemistry as a Tool for Redox‐Based Bio‐Information Processing
Source: Adv Sci (Weinh). 2025 Aug 22;12(36):e10184. doi: 10.1002/advs.202510184 (PMC12463114; doi:10.1002/advs.202510184)
Supplement: Supplementary file 1 — Supporting Information [file ADVS-12-e10184-s001.pdf]

ADVANCED SCIENCE
Open Access

Supporting Information

for *Adv. Sci.*, DOI 10.1002/advs.202510184

Electrochemistry as a Tool for Redox-Based Bio-Information Processing

Eunkyoung Kim, Chen-yu Chen, Fauziah Rahma Zakaria, Dana Motabar, Mijeong Kang, Deanna L. Kelly, Alessandra Napolitano, William E. Bentley and Gregory F. Payne**

Supporting Information

ELECTROCHEMISTRY AS A TOOL FOR REDOX-BASED BIO-INFORMATION PROCESSING

Eunkyoung Kim, Chen-yu Chen, Fauziah Rahma Zakaria, Dana Motabar, Mijeong Kang,
Deanna L. Kelly, Alessandra Napolitano, William E. Bentley, Gregory F. Payne

Table S1. Electrochemical mediated probing methods for redox-based bio-information processing

Redox-based information	Target molecule	Mediator		Electrode	Technique	Findings	Reference
		Oxidizing	*Reducing*				
**Redox Activity**	Melanin	Fc, AS, ABTS, Ir^{3+}	Ru^{3+}, PYO, PQ	Gold, ITO	CV, CC	Reversible redox activity of melanin	[1-5]
	Humic acids, lignin	Fc	Ru^{3+}	ITO	CV, CC	Reversible redox activity of humic acids, lignin	[6]
**Redox Interaction**	Paraquat-dehydroascorbate	Dehydroascorbate	PQ	Gold	CV, SEC	Dehydroascorbate's quenching of PQ radical	[7]
	Drugs-melanin	Acetaminophen, Clozapine	Mel-Chit/ Ru^{3+}	Gold	CV	Redox cycling of analgesic acetaminophen and antipsychotic clozapine with melanin	[8]
**Chemical Information**	Single amino acid variation (SAV); Proline-residue	Ru_{bpy}^{3+}		ITO	CV, ECL	ECL measurement of proline-relevant SAV	[9]
	Antibody structure; Cys-residue	Fc, Ir^{3+}		GC, ITO	CV, CC	*Electrochemical measurement of cysteine-relevant antibody structure	[10, 11]
	Oxidative stress (Reducing capacity)	Ir^{3+}		Gold	CV, CC, SEC	*Electrochemical measurement of reducing capacity of serum	[12, 13]
	Bacterial infection	Fc/ Cat-Chit	PYO	Gold	CV, CC	Electrochemical measurement of bacterial infection (*P. aeruginosa*)	[14]
**Electrogenetic Actuation**	SoxR	Fcn, PYO		Gold	CV, CC	Mediated electrogenetic activation with SoxR	[15]
	OxyR	AS	H_2O_2	Gold	CV, CC	Mediated electrogenetic activation with OxyR	[16]

- **Mediators:** Ferrocene dimethanol (Fc), $K_3Fe(CN)_6$ (Fcn), $Ru(NH_3)_6Cl_3$ (Ru^{3+}), Acetrosyringone (AS), Paraquat (PQ), 2,2'-azino-bis(3-ethylbenzothiazoline-6-sulfonic acid (ABTS), Tris(bipyridine)ruthenium(II) chloride (Ru_{bpy}^{3+}), K_3IrCl_6 (Ir^{3+}), Pyocyanin (PYO),
- **Modified film**: Catechol-modified chitosan (Cat-Chit), Melanin entrapped chitosan (Mel-Chit)
- **Electrode**: Glassy carbon (GC), Indium tin oxide (ITO)
- **Technique**: Cyclic voltammetry (CV), Chronocoulometry (CC), Spectroelectrochemistry (SEC), Electrochemiluminescence (ECL)

***To overcome the electrode fouling;**

1. The glassy carbon electrode is polished before each electrochemical measurement.[10]
2. The indium tin oxide (ITO) electrode is used because ITO has the greater resistance to the electrode fouling by antibody.[11]
3. The gold electrode is electrochemically cleaned in sulfuric acid to remove any remaining surface contamination after assaying serum.[12]

Table S2. Characteristics of common working electrodes

Properties	Carbon (Glassy Carbon, GC)	Gold (Au)	Indium Tin Oxide (ITO)
**Conductivity**	Good	Excellent	Good
**Potential window**	Wide (especially cathodic);<br>Good anodic in non-aqueous	Good;<br>Anodic limited by surface oxidation	Wide (especially anodic);<br>Cathodic limited by reduction of ITO
**Optical property**	Opaque	Opaque	Transparent in visible light
**Fouling resistance**	Good<br>Still adsorb organic molecules	Moderate (because oxide formation and thiol-affinity)	Good
**Electrode cleaning**	Polish	Polish, chemical treatment (e.g., piranha solution)	Chemical treatment (e.g., methanol, isopropanol)

References

[1] E. Kim, W. T. Leverage, Y. Liu, L. Panzella, M. L. Alfieri, A. Napolitano, W. E. Bentley, G. F. Payne, "Paraquat–Melanin Redox-Cycling: Evidence from Electrochemical Reverse Engineering", *ACS Chem. Neurosci.,* **2016**, *7* (8), 1057, https://doi.org/10.1021/acschemneuro.6b00007.

[2] E. Kim, M. Kang, T. Tschirhart, M. Malo, E. Dadachova, G. Cao, J.-J. Yin, W. E. Bentley, Z. Wang, G. F. Payne, "Spectroelectrochemical reverse engineering DemonstratesThat Melanin's redox and radical scavenging activities are linked", *Biomacromolecules,* **2017**, *18* (12), 4084, https://doi.org/https://doi.org/10.1021/acs.biomac.7b01166.

[3] M. Kang, E. Kim, Z. Temoçin, J. Li, E. Dadachova, Z. Wang, L. Panzella, A. Napolitano, W. E. Bentley, G. F. Payne, "Reverse Engineering to Characterize Redox Properties: Revealing Melanin's Redox Activity through Mediated Electrochemical Probing", *Chem. Mater.,* **2018**, *30* (17), 5814, https://doi.org/10.1021/acs.chemmater.8b02428.

[4] E. Kim, Z. Wang, J. W. Phua, W. E. Bentley, E. Dadachova, A. Napolitano, G. F. Payne, "Enlisting electrochemistry to reveal melanin's redox-related properties", *Mater. Adv.,* **2024**, *5* (8), 3082, https://doi.org/10.1039/D3MA01161E.

[5] E. Kim, L. Panzella, R. Micillo, W. E. Bentley, A. Napolitano, G. F. Payne, "Reverse Engineering Applied to Red Human Hair Pheomelanin Reveals Redox-Buffering as a Pro-Oxidant Mechanism", *Sci. Rep.,* **2015**, *5*, 18447, https://doi.org/10.1038/srep18447.

[6] E. Kim, Y. Liu, C. J. Baker, R. Owens, S. Xiao, W. E. Bentley, G. F. Payne, "Redox-cycling and H2O2 generation by fabricated catecholic films in the absence of enzymes", *Biomacromolecules,* **2011**, *12* (4), 880, https://doi.org/10.1021/bm101499a.

[7] Z. Zhao, E. Kim, D. Motabar, W. E. Bentley, G. F. Payne, "Spectroelectrochemical Network Measurements for Redox Bioelectronics", *Chem Mater,* **2023**, *35* (3), 976, https://doi.org/10.1021/acs.chemmater.2c02707.

[8] Z. Temoçin, E. Kim, J. Li, L. Panzella, M. L. Alfieri, A. Napolitano, D. L. Kelly, W. E. Bentley, G. F. Payne, "The Analgesic Acetaminophen and the Antipsychotic Clozapine Can Each Redox-Cycle with Melanin", *ACS Chem. Neurosci.,* **2017**, *8* (12), 2766, https://doi.org/10.1021/acschemneuro.7b00310.

[9] E. Kim, C.-Y. Chen, M. J. Chu, M. Hamstra, W. E. Bentley, G. F. Payne, "Proline-Selective Electrochemiluminescence Detecting a Single Amino Acid Variation Between A1 and A2 β-Casein Containing Milks", *Adv. Sci.,* **2024**, *12*, 2411956, https://doi.org/10.1002/advs.202411956.

[10] D. Motabar, E. Kim, J. Li, Z. Zhao, T. Mouchahoir, D. T. Gallagher, J. E. Schiel, M. Garige, C. Sourbier, G. F. Payne, W. E. Bentley, "Detecting features of antibody structure through their mediator-accessible redox activities", *Nat. Chem. Biol.,* **2024**, *21* (2), 291, https://doi.org/10.1038/s41589-024-01778-z.

[11] C. Y. Chen, E. Kim, F. R. Zakaria, M. J. Chu, B. Wu, G. F. Payne, W. E. Bentley, "3D Printed Spectroelectrochemical Platform for Redox-Based Bioelectronics", *Small Methods,* **2025**, 2401843.

[12] M. Kang, E. Kim, T. E. Winkler, G. Banis, Y. Liu, C. A. Kitchen, D. L. Kelly, R. Ghodssi, G. F. Payne, "Reliable clinical serum analysis with reusable electrochemical sensor: Toward point-of-care measurement of the antipsychotic medication clozapine", *Biosens Bioelectron,* **2017**, *95*, 55, https://doi.org/10.1016/j.bios.2017.04.008.

[13] E. Kim, T. E. Winkler, C. Kitchen, M. Kang, G. Banis, W. E. Bentley, D. L. Kelly, R. Ghodssi, G. F. Payne, "Redox Probing for Chemical Information of Oxidative Stress", *Anal. Chem.,* **2017**, *89* (3), 1583, https://doi.org/http://dx.doi.org/10.1021/acs.analchem.6b03620.

[14] E. Kim, T. Gordonov, W. E. Bentley, G. F. Payne, "Amplified and in Situ Detection of Redox-Active Metabolite Using a Biobased Redox Capacitor", *Anal. Chem.,* **2013**, *85*, 2102, https://doi.org/10.1021/ac302703.

[15] T. Tschirhart, E. Kim, R. McKay, H. Ueda, H.-C. Wu, A. E. Pottash, A. Zargar, A. Negrete, J. Shiloach, G. F. Payne, W. E. Bentley, "Electronic control of gene expression and cell behaviour in Escherichia coli through redox signalling", *Nat Commun,* **2017**, *8*, 14030, https://doi.org/10.1038/ncomms14030.

[16] F. R. Zakaria, C. Y. Chen, J. Y. Li, S. Wang, G. F. Payne, W. E. Bentley, "Redox active plant phenolic, acetosyringone, for electrogenetic signaling", *Sci. Rep.,* **2024**, *14* (1), 9666, https://doi.org/10.1038/s41598-024-60191-7.
